# *SCS3* and *YFT2* Link Transcription of Phospholipid Biosynthetic Genes to ER Stress and the UPR

**DOI:** 10.1371/journal.pgen.1002890

**Published:** 2012-08-23

**Authors:** Robyn D. Moir, David A. Gross, David L. Silver, Ian M. Willis

**Affiliations:** 1 Department of Biochemistry, Albert Einstein College of Medicine, Bronx, New York, United States of America; 2 Signature Research Program in Cardiovascular and Metabolic Disorders, Duke–NUS Graduate Medical School Singapore, Singapore, Singapore; 3 Department of Systems and Computational Biology, Albert Einstein College of Medicine, Bronx, New York, United States of America; University of California San Francisco, United States of America

## Abstract

The ability to store nutrients in lipid droplets (LDs) is an ancient function that provides the primary source of metabolic energy during periods of nutrient insufficiency and between meals. The Fat storage-Inducing Transmembrane (FIT) proteins are conserved ER–resident proteins that facilitate fat storage by partitioning energy-rich triglycerides into LDs. FIT2, the ancient ortholog of the *FIT* gene family first identified in mammals has two homologs in *Saccharomyces cerevisiae* (*SCS3* and *YFT2*) and other fungi of the *Saccharomycotina* lineage. Despite the coevolution of these genes for more than 170 million years and their divergence from higher eukaryotes, *SCS3*, *YFT2*, and the human *FIT2* gene retain some common functions: expression of the yeast genes in a human embryonic kidney cell line promotes LD formation, and expression of human *FIT2* in yeast rescues the inositol auxotrophy and chemical and genetic phenotypes of strains lacking *SCS3*. To better understand the function of *SCS3* and *YFT2*, we investigated the chemical sensitivities of strains deleted for either or both genes and identified synthetic genetic interactions against the viable yeast gene-deletion collection. We show that *SCS3* and *YFT2* have shared and unique functions that connect major biosynthetic processes critical for cell growth. These include lipid metabolism, vesicular trafficking, transcription of phospholipid biosynthetic genes, and protein synthesis. The genetic data indicate that optimal strain fitness requires a balance between phospholipid synthesis and protein synthesis and that deletion of *SCS3* and *YFT2* impacts a regulatory mechanism that coordinates these processes. Part of this mechanism involves a role for *SCS3* in communicating changes in the ER (e.g. due to low inositol) to Opi1-regulated transcription of phospholipid biosynthetic genes. We conclude that *SCS3* and *YFT2* are required for normal ER membrane biosynthesis in response to perturbations in lipid metabolism and ER stress.

## Author Summary

The ability to form lipid droplets is a conserved property of eukaryotic cells that allows the storage of excess metabolic energy in a form that can be readily accessed. In adipose tissue, the storage of excess calories in lipid droplets normally protects other tissues from lipotoxicity and insulin resistance, but this protection is lost with chronic over-nutrition. The FAT storage-inducing transmembrane (FIT) proteins were recently identified as a conserved family of proteins that reside in the lipid bilayer of the endoplasmic reticulum and are implicated in lipid droplet formation. In this work we show that specific functions of the FIT proteins are conserved between yeast and humans and that *SCS3* and *YFT2*, the yeast homologs of mammalian *FIT2*, are part of a large genetic interaction network connecting lipid metabolism, vesicle trafficking, transcription, and protein synthesis. From these interactions we determined that yeast strains lacking *SCS3* and *YFT2* are defective in their response to chronic ER stress and cannot induce the unfolded protein response pathway or transcription of phospholipid biosynthetic genes in low inositol. Our findings suggest that the mammalian *FIT* genes may play an important role in ER stress pathways, which are linked to obesity and type 2 diabetes.

## Introduction

Eukaryotic cells store neutral lipids (triglycerides, TGs and steryl esters, SEs) in cytoplasmic lipid droplets (LDs) surrounded by a monolayer of phospholipids and associated proteins [1]. These sub-cellular structures are mobile, dynamic organelles that grow and shrink depending on metabolic conditions [2]–[6]. Lipid droplets serve as the principal reservoirs for storing cellular energy and provide the building blocks for membrane lipids [1], [3], [4]. Stored lipid is accessed in a regulated fashion to provide energy through β-oxidation of fatty acids and substrates for the synthesis of other important cellular molecules, such as membrane phospholipids and eicosanoids. In addition, the products of TG hydrolysis, namely diacylglycerol (DAG) and free fatty acids, are important regulators of cellular signaling either directly or after subsequent metabolism (e.g. to phosphatidic acid, PA; or ceramide) [7], [8]. LDs also play a central role in cholesterol homeostasis. The storage and release of sterols from LDs can alter the physical properties of membranes, affect the levels of circulating free cholesterol and contribute to the synthesis of steroid hormones and bile acids [1], [9]. Despite broad recognition of the importance of LDs in cellular metabolism and in diseases associated with excessive lipid storage (e.g. obesity, type II diabetes, atherosclerosis and fatty liver disease), their biogenesis, regulatory mechanisms and the nature of their interactions with other organelles are still largely unknown.

Fat storage-Inducing Transmembrane proteins 1 & 2 (FITM1/FIT1 and FITM2/FIT2) were identified as unique, evolutionarily conserved ER-resident proteins that affect the partitioning of TG into LDs [10]. The FIT proteins exhibit different tissue distributions with FIT1 highly expressed skeletal muscle, lower levels in heart, and with FIT2 broadly distributed and most abundant in adipose tissue. Studies in both cultured cells and mice have shown that overexpression of the FIT genes promotes the accumulation of LDs [10], [11]. Importantly, these changes occur without inducing TG biosynthesis or inhibiting lipolysis. Conversely, knockdown of *FIT* gene expression decreases LD production in an adipocyte differentiation cell culture model and in zebrafish [10]. Thus, the data suggest that FIT protein function is critical for LD formation and can drive this process without affecting TG biosynthesis or turnover [10].

Budding yeast contains two *FIT2* gene homologs, *SCS3* and *YFT2*, that are readily identified as the only homologous genes in *S. cerevisiae* in BLAST searches using the full-length human protein as a query (with E values of 3.7E-5 and 5.5E-7, respectively) [10]. The majority of this sequence conservation occurs with the predicted transmembrane domains. In an experimentally-constrained global topology study of the yeast proteome, Scs3 and Yft2 (like most polytopic yeast membrane proteins) were predicted to have cytosolic N and C termini and therefore an even-number of transmembrane (TM) helices [12]. Consistent with this, a topological analysis of the murine FIT proteins demonstrated the cytoplasmic localization of both termini and a six-transmembrane domain organization [13]. The mechanism by which the mammalian FIT proteins mediate their effects on LD production has not been determined. However, recent work has found that the FIT proteins bind neutral lipids (TG and DAG) and that the extent and/or affinity of this interaction correlates with LD size [14]. Notably, a gain-of-function mutation (FLL(157-9)AAA) in the conserved TM4 domain of mouse FIT2 which increases TG binding and LD size has also been shown to alter the conformation of a cytoplasmic loop connecting TM domains 2 and 3 [13]. Thus, changes in the conformation of FIT proteins, potentially induced by TG binding, may influence the size of LDs [14].

The metabolic pathways leading to the formation of neutral lipids and the hydrolytic reactions catalyzing their mobilization are conserved between yeast and mammalian cells [15]–[17]. The major classes of glycerophospholipids in yeast membranes and their biosynthetic pathways are also conserved [15], [18]. Yeast mutants with defects in TG synthesis, storage and catabolism have been identified and provide valuable models for understanding disease phenotypes such as obesity, lipodystrophy and lipotoxicity [19]–[21]. These studies support the use of yeast (and other model organisms) to better understand the biogenesis and function of LDs [22]–[24]. To this end, two visual screens of the viable yeast gene-deletion collection have been conducted to identify mutants defective in LD number and morphology. Both screens discovered numerous genes involved in LD biology and implicated functions that were not previously associated with this process [22], [23]. However, a comparison of the genes reported in these studies reveals a limited overlapping set. This suggests that additional effectors of LD biogenesis and function remain to be identified. Consistent with this view, re-screening of the deletion collection on defined minimal media uncovered several gene-deletions that form supersized (>1 µm diameter) LDs [25].

We have initiated studies in yeast to identify chemical and synthetic genetic phenotypes associated with deletions of the mammalian *FIT* gene homologs, *SCS3* and *YFT2*. Despite the distant evolutionary relationship between these genes in fungi, the data show that *SCS3* and *YFT2* have shared as well as unique functions. Common functions are also demonstrated between the proteins in yeast and human systems. However, in contrast to *FIT* gene knockdown experiments in higher eukaryotes, deletion of *SCS3* and/or *YFT2* does not noticeably impact the number or size of LDs. A genetic network centered on *SCS3* and *YFT2* is presented that identifies a multitude of aggravating and alleviating interactions that connect major biosynthetic processes critical for cell growth. We find that the functions of *SCS3* and *YFT2* relate lipid metabolism and signaling with transcription of phospholipid biosynthetic genes and protein synthesis. More specifically, the data show that optimal strain fitness requires a balance between phospholipid synthesis and protein synthesis and suggest that the function of Scs3 and Yft2 impacts a regulatory response that serves to coordinate these processes.

## Results/Discussion

### Evolution of *SCS3* and *YFT2* and phenotypic analysis of gene-deletion strains

Prior to the discovery of the *FIT* genes in mammals [10], *YFT2* was an uncharacterized open reading frame (YDR319C) whose relationship (structural or functional) to *SCS3* was not appreciated. Consequently, none of the studies conducted to date on *SCS3* have taken into account its potential functional redundancy with *YFT2*. Phylogenetic evidence from 42 sequenced fungal genomes [26] indicates that *YFT2* arose by a segmental duplication of *SCS3* (Figure S1). Based on the distribution of *YFT2* in these fungi, this segmental duplication preceded both the whole-genome duplication that characterizes many *Saccharomyces* species and their divergence from *Candida* species that decode CTG as serine instead of leucine (Figure S1). These observations indicate that *SCS3* and *YFT2* have been maintained in the *Saccharomycotina* lineage for >170 million years [26] and imply that each gene confers a selective advantage to those organisms. This notion is supported by genetic interaction data for duplicate gene pairs that have been retained following the genome-wide duplication [27]. The selective advantage to yeast of retaining *SCS3* and *YFT2* could reflect differences in their patterns of expression and/or functional differences. Indeed, differences in expression following certain chemical or genetic perturbations are evident using SPELL to search a compendium of ∼2400 microarray experiments [28]. To search for functional differences, we screened the *scs3*Δ and *yft2*Δ single deletion strains and the *scs3*Δ *yft2*Δ double deletion strain for growth defects under a wide variety of conditions [29]. The screen which included 25 different chemicals and drugs, various carbon sources, growth temperatures and osmolarity conditions revealed surprisingly few phenotypes.

**Figure 1:**
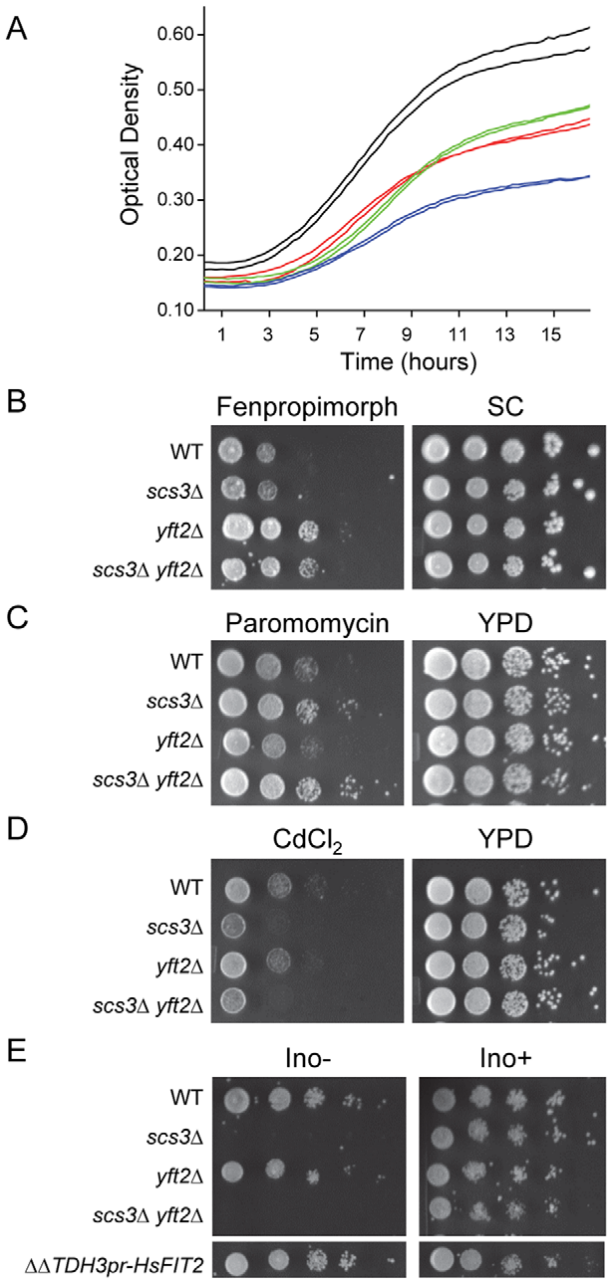
Characterization of *SCS3* and *YFT2* gene-deletion phenotypes. (A) Effect of cerulenin on the growth of *SCS3* and *YFT2* gene-deletion strains. Strains were inoculated in duplicate into YPD containing cerulenin (3.2 µM) and grown with shaking at 30°C. Optical density was recorded using a Bioscreen Analyzer C at 15 min intervals. Cerulenin inhibition of fatty acid synthesis limited yeast growth and viability resulting in low cell density at saturation and slow apparent doubling times during log phase growth: Wt, 233±9 min (black); *scs3*Δ, 309±75 min (green); *yft2*Δ, 197±6 min (red); *scs3*Δ *yft2*Δ, 1088±2 min (blue). Doubling times of all strains in YPD+DMSO averaged 92±4 min and all strains reached a comparably high cell density at saturation (data not shown). (B–D) Plate phenotypes of *scs3*Δ, *yft2*Δ and double mutant (ΔΔ) strains. Equal cell numbers from 10-fold serial dilutions were spotted onto media containing drugs or inorganic compounds and scored for growth after 2 to 3 days at 30°C. Rich medium (YPD) or synthetic complete medium (SC) contained drug delivery solvent or (B) fenpropimorph (0.1 µM), (C) paromomycin (4 mg/ml), (D) CdCl_2_ (25 µM). (E) Serial dilutions of *scs3*Δ, *yft2*Δ and double mutant (ΔΔ) strains were spotted on defined synthetic medium in the presence or absence of inositol (75 µM). Complementation of the Ino- phenotype in the *scs3*Δ *yft2*Δ strain is shown by an integrated copy of the human FIT2 gene under the control of the yeast *TDH3* promoter. Images are from non-contiguous regions of the same plates.

Cerulenin is a specific inhibitor of fatty acid synthase [30]. Gene deletions that are hypersensitive to cerulenin include *INO2* and *INO4*, which together encode the transcriptional activator that binds inositol-responsive (UAS_INO_) elements and drives the expression of phospholipid biosynthetic enzymes and other lipid metabolic proteins [31], [32]. Strains lacking either *SCS3* or *YFT2* or both genes were hypersensitive to cerulenin as indicated by the reduced cell density at saturation (Figure 1a). In addition, the *scs3*Δ strain and the double deletion strain exhibited longer apparent doubling times during exponential growth (Figure 1a). These properties show that deletion of *SCS3* and/or *YFT2* exacerbates the limited supply of fatty acids available via *de novo* synthesis. In addition to this shared phenotype, we found other phenotypes that were specific for strains lacking either *SCS3* or *YFT2*, consistent with these genes having unique functions. For example, strains deleted for *YFT2* were resistant to fenpropimorph, which acts on 8,7-sterol isomerase (Erg2) and sterol-14 reductase (Erg24) to inhibit ergosterol biosynthesis (Figure 1b). This resistance was not seen in the *scs3*Δ strain and was not enhanced in the *scs3*Δ *yft2*Δ double mutant indicating that *SCS3* function does not contribute to this phenotype (Figure 1b). In the absence of exogenous ergosterol, resistance to fenpropimorph has been reported to occur by a gain of function mutation in the plasma membrane H+-pantothenate symporter, *FEN2*, or by loss of function mutations in *FEN1*, an ER-localized fatty acid elongase involved in sphingolipid biosynthesis [33]. Accordingly, deletion of *YFT2* may alter the activity of the membrane-localized Fen1 or Fen2 enzymes or negatively impact sphingolipid biosynthesis.

Phenotypes associated specifically with deletion of *SCS3* included cadmium hypersensitivity, paromomycin resistance and inositol auxotrophy (Figure 1c–1e). Interestingly, the resistance to paromomycin does not reflect a general resistance of *scs3*Δ strains to aminoglycoside antibiotics (e.g. G418, data not shown) but relates *SCS3* function to increased translational fidelity based on the properties of a single base substitution within yeast 18S rRNA that also confers this phenotype [34]. Deletion of *SCS3* increased the toxicity of cadmium ions. Although the mechanism of this toxicity is not thoroughly understood, cadmium is known to cause lipid peroxidation and like many metals, affects membrane fluidity [35]. Moreover, the uptake and detoxification of cadmium is strongly influenced by plasma membrane and vacuolar transporters [35]. These observations suggest that the cadmium hypersensitivity of *scs3*Δ strains results from an accumulation of metal ion, potentially due to altered membrane composition and/or transporter function (see below). Inositol auxotrophy has been associated with deletion of *SCS3* since the gene was first cloned [36]. Recently, the Ino- phenotype of the *scs3*Δ strain was attributed to decreased levels of inositol-3-phosphate synthase (Ino1), the rate-limiting enzyme in the synthesis of phosphatidylinositol (PI) [37]. This phenotype is not shared by strains lacking *YFT2* (Figure 1e). However, the function of Scs3 that allows growth in the absence of inositol is conserved in human *FIT2*: A genomic copy of the human *FIT2* gene expressed from the yeast *TDH3* promoter complemented the inositol auxotrophy of *scs3*Δ (Figure 1e) as well as other *scs3*-specific phenotypes (e.g. paromomycin resistance, data not shown, and certain synthetic genetic phenotypes, Figure S2a, discussed below). Importantly, expression of human *FIT2* in yeast did not activate the UPR, a measure of ER stress, Figure S2b). Thus, the complementation mutant phenotypes by human *FIT2* indicates that *SCS3* is related to the ancestral *FIT* gene that is broadly distributed in eukaryotes and supports the phylogenetic conclusion that *YFT2* was derived by an ancient segmental duplication of *SCS3*.

### Scs3 and Yft2 can induce lipid droplet formation in human cells

**Figure 2:**
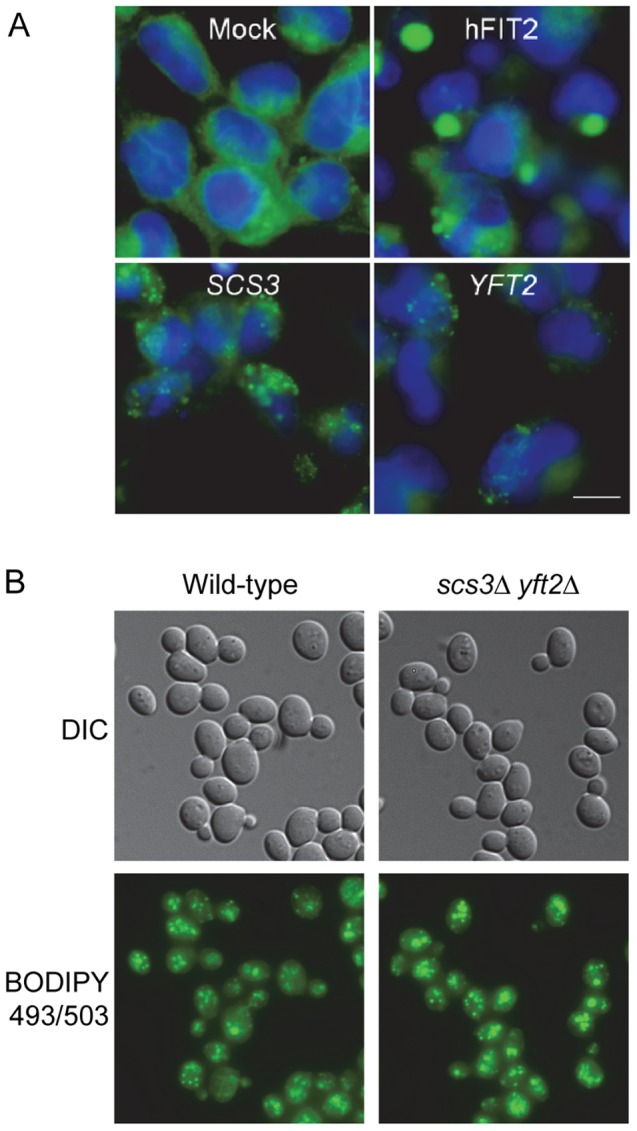
Lipid droplets in human cells expressing *SCS3* and *YFT2* and in yeast gene-deletion strains. (A) HEK293 (human embryonic kidney cells) were transfected with empty pcDNA3.1 vector (Mock), or expression vectors for *H. sapiens* FIT2 (hFIT2), *S. cerevisiae SCS3*, or *S. cerevisiae YFT2*. Cells were stained for lipid droplets (LDs, green) using BODIPY 493/503 and nuclei using Hoechst 33342 (blue). Images are representative of two different experiments. Scale bar, 10 µm. (B) Early log phase cultures of a wild-type yeast strain and a strain lacking both *SCS3* and *YFT2* were fixed with formaldehyde and stained with BODIPY 493/503 (0.5 µg/ml) in PBS. Fluorescence was recorded using optical sectioning (100× magnification) and the images were combined into a single projection. Analysis of ∼300 cells of each strain yielded 7.1±2.5 and 7.9±3.6 LDs/cell for the wild-type and double mutant strains, respectively. Similar numbers were obtained for the single mutant strains.

Given the complementation of mutant phenotypes by human *FIT2* in yeast, we used the fluorescent LD-specific dye BODIPY 493/503 to determine whether either *SCS3* or *YFT2* could drive the production of LDs in human embryonic kidney (HEK293) cells. Remarkably, transient expression of either *SCS3* or *YFT2* under the control of the CMV promoter induced the appearance of LDs in HEK293 cells similar to overexpression of the human and mouse *FIT* genes (Figure 2a) [10]. Notably, the size of the LDs induced by the yeast proteins was smaller than for human FIT2 (Figure 2a). Recently, Gross et al [14] showed that droplet size in this assay correlates with the affinity and/or extent of FIT protein binding to TG in vitro. Thus, the yeast proteins may have a reduced capacity for TG binding compared to human FIT2. Overall, the results are consistent with the view that an ancient function involved in stimulating LD formation has been conserved in *SCS3*, *YFT2* and human *FIT2*. Based on these findings and the ability of *FIT2* knock-downs to dramatically diminish the appearance of LDs in NIH 3T3 L1 adipocytes and zebrafish [10], we anticipated that a yeast strain lacking either *SCS3* or *YFT2*, or both genes, would exhibit a LD phenotype. Contrary to this expectation, fluorescence microscopy of log phase wild-type, *scs3*Δ, *yft2*Δ and *scs3*Δ *yft2*Δ cells stained with BODIPY 493/503 showed no differences in the number of LDs (Figure 2b and data not shown). Similarly, no differences were found between the same strains at stationary phase although the number of droplets increased relative to log phase (∼13 versus 7–8 LDs/cell, in either fixed or unfixed cell preparations, data not shown). An examination of LD formation in the *scs3*Δ *yft2*Δ strain under several other growth conditions including low inositol and in oleate-containing media also did not reveal any phenotype (Figure S3a and S3b). Taken at face value, these results suggest that *SCS3* and *YFT2* do not play a role in LD biogenesis in yeast. While this may be the case, the properties of *FIT* proteins in other organisms [10], [11], [38], the complementation by human FIT2 in yeast (Figure 1e and Figure S2a), the ability of *SCS3* and *YFT2* to induce LDs in human cells (Figure 2a) and the extensive genetic interactions linking *SCS3* and *YFT2* with lipid metabolism and transport in yeast (see below), suggest that more complex interpretations should be considered: Yeast may possess alternative mechanisms for storing neutral lipids in droplets. Thus, the absence of one mechanism due to deletion of *SCS3* and *YFT2* may be compensated by the presence of another. Differences in the size of LDs between *S. cerevisiae* and organisms where *FIT* gene-dependent changes have been observed could also be important: The average diameter of LDs in yeast ranges between 0.35–0.45 µm compared to 1.1 µm in *C. parapsilosis* and up to 100 µm in higher eukaryotes [38]–[40].

### Digenic and trigenic interactions of *SCS3* and *YFT2* in budding yeast

**Figure 3:**
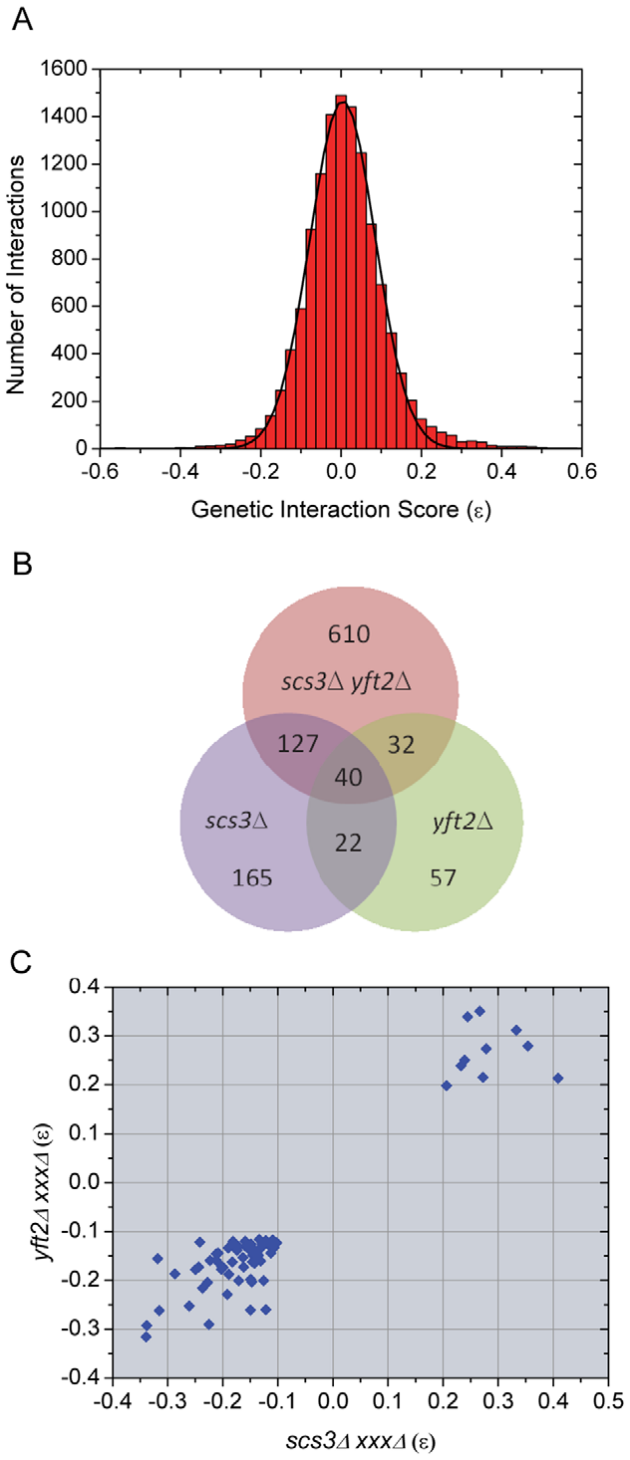
Evaluation and overlap of SGA data from screens of *scs3*Δ, *yft2*Δ, and *scs3*Δ *yft2*Δ strains. (A) Genetic interaction scores (ε values) for all strains in all screens show a normal distribution centered on zero (no bias in the multiplicative model). Epsilon values determined for each array gene-deletion in combination with *scs3*Δ or *yft2*Δ or *scs3*Δ *yft2* **Δ** were combined, binned (bin size = 0.025) and fit to a Gaussian function. The fitted curve was centered at 0.004±6.7E-4 with σ = 0.08 and an adjusted *r^2^* = 0.997. Similar quality fits were obtained when data from individual query screens were analyzed separately. (B) Overlapping genetic interactions between *SCS3* and *YFT2*. The Venn diagram shows the intersection of genes exhibiting aggravating or alleviating interactions in screens with the three query strains. The stringent criteria for inclusion in this comparison required a size difference between query and control screens of >40 pixels and a *p* value<0.01 (see Material and Methods). Interactions with 221 genes were found in two or more screens. (C) Pairwise comparison of genetic interaction scores within the set of 62 genetic interactions identified in screens of the individual *SCS3* and *YFT2* deletion strains (see panel b). Similar comparisons for the other two pairwise combinations are shown in Figure S4.

To further investigate the unique and shared functions of *SCS3* and *YFT2* and to uncover functional relationships with other genes and processes, we conducted synthetic genetic array (SGA) analysis using query strains deleted for either or both genes (see Materials and Methods). Part of our rationale for including the *scs3*Δ *yft2*Δ double mutant strain as a query was to identify genetic interactions associated with the conserved redundant function that drives LD formation in human cells (Figure 2a). Colony sizes of the double and triple mutant strains generated with the three query strains were scored by computer-based analysis of digital images and used to determine strain fitness by comparison to a reference set of images from control screens. Genetic interactions were quantitatively scored as the difference between the observed and expected fitness, determined using a multiplicative model [41] for each double or triple mutant strain (see Materials and Methods). As a measure of the quality of the data, we determined that the colony sizes of 93% of the strains (∼4000 out of 4292 strains in each screen) had coefficients of variation less than 22.5% with an average of 12±7% for the 14504 *his3*Δ*::kanMX* control strains that were used for colony size normalization (see Materials and Methods). In addition, we examined the genetic interaction score (ε values) for all strains in each screen and found them to be normally distributed and centered on zero indicating no bias in the multiplicative model (Figure 3a). Guided by a recent large scale study [42], we set a stringent threshold for differences in colony size and p value (≥40 pixels and ≤0.01, respectively) and examined the genetic interaction overlap in pairwise comparisons of the three screens (Figure 3b). Of the 354 interactions with *SCS3* that met the preceding criteria, ∼50% were identified with the double mutant query. A similar percentage of interactions with the double mutant query were also identified among the 151 interactions with *YFT2*. Overall, 221 interactions were shared in screens involving two or more of the query strains indicating a high probability that the majority of these are true positive interactions. Consistent with the view that *SCS3* and *YFT2* retain some redundant function (see above), we found that the total number of interactions increased significantly using the double mutant query (Figure 3b). This reflects the loss of buffering capacity for the redundant function when both genes are deleted. Similar observations have been made in screens of the partially redundant G1 cyclins, *CLN1* and *CLN2* [43] and other duplicate gene pairs [27].

Pairwise comparisons of genetic interaction scores (ε values) among the 221 “true positives” revealed a sharp cutoff for aggravating and alleviating interactions at −0.1 and 0.2, respectively, in the single mutant query screens (Figure 3c). The same sharp cutoffs were also seen in comparisons of the single versus double mutant screens although the boundary for aggravating effects involving the double mutant query was slightly higher at −0.05 (Figure S4). Given the large number of interactions in the three screens (Figure 3b), we set a high stringency cutoff of −0.12 for aggravating interactions, as reported by the Costanzo et al., [42] and 0.2 for alleviating interactions (Figure 3c). This produced a set of 636 interactions with known (verified) or uncharacterized genes (Table S1). During this analysis, we found that our screens identified numerous genes annotated as “dubious ORFs” in SGD that overlapped the coding or promoter regions of known genes that were also on the array. These dubious ORFs provided an additional way of testing the reproducibility of the SGA data: We compiled a list of all the dubious ORFs that met our three criteria (pixel size, *p* value and ε score) in at least one of the three screens and then compared their ε values for all three queries with the corresponding values for the overlapping verified genes. The scores were highly correlated (Pearson's r = 0.75, Figure S5).

### Unique, shared, and opposing interactions among *scs3*Δ, *yft2*Δ, and *scs3*Δ *yft2*Δ strains

**Figure 4:**
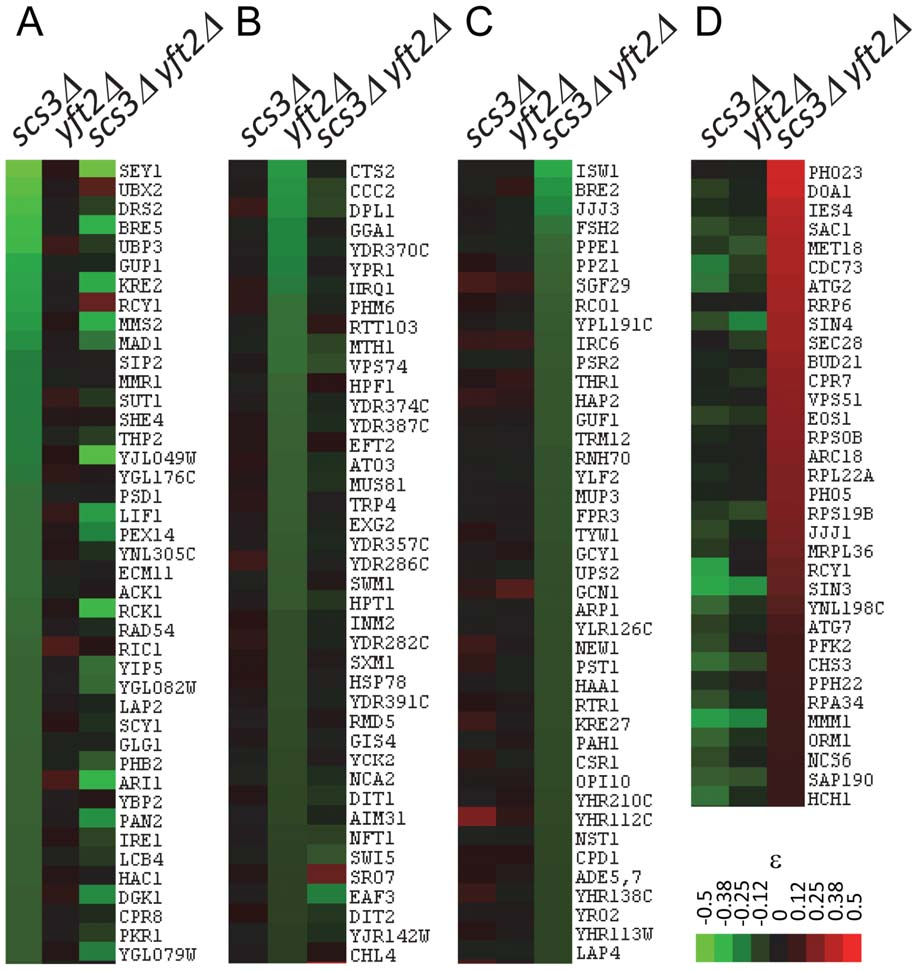
Representative genetic interactions obtained in SGA screens with *SCS3* and *YFT2* gene-deletion strains. Clustergrams show interactions representing unique, shared and antagonizing functions of *SCS3* and *YFT2* in the set of 636 genes defined by stringent selection criteria (see text). (A) Aggravating interactions identified for *SCS3* but not *YFT2* (ε>−0.05). (B) Aggravating interactions identified for *YFT2* but not *SCS3* (ε>−0.05). (C) Aggravating interactions identified for the *SCS3 YFT2* double mutant but not for either gene individually (ε>−0.05). (D) Alleviating interactions identified for the *SCS3 YFT2* double mutant where the individual genes showed an aggravating or no interaction (ε<0).

Synthetic genetic interactions reveal functional relationships between genes [44], [45]. Based on the gene-specific and shared phenotypes described above (Figure 1), we expected our SGA analysis to identify sets of genetic interactions that reflect the unique and common functions of *SCS3* and *YFT2*. Indeed, we identified many interactions with one or the other gene as well as interactions that required deletion of both genes (Figure 4a–4c). These genetic data provide strong support for the existence of unique and shared functions for *SCS3* and *YFT2* in yeast. In addition, we identified a fourth class of interactions where opposing phenotypes were found for single gene deletions versus the double deletion strain: Deletions of numerous genes showed a strong aggravating phenotype in combination with *scs3*Δ and/or *yft2*Δ single mutants but a strong alleviating phenotype with the *scs3*Δ *yft2*Δ double mutant (Figure 4d). In other cases, aggravating phenotypes obtained with each single mutant were genetically suppressed (i.e. ε∼0 in the double mutant). Since the fitness of the *scs3*Δ, *yft2*Δ and *scs3*Δ *yft2*Δ strains is very similar to wild-type (ε = 0.96, 0.96 and 0.89, respectively, versus 1.0 for the wild-type strain, see Materials and Methods), the interpretation of suppressing or alleviating interactions with these strains primarily involves rescuing the poor fitness of the respective deletion strains on the array. Noteworthy examples are provided by *SAC1* and *CHS3*. Sac1 is a phosphatidylinositol phosphate (PtdInsP) phosphatase that is localized to the ER and Golgi and functions in protein trafficking, secretion and cell wall maintenance. Deletion of *SAC1* dramatically and selectively increases the level of PtdIns(4)P (8–12 fold) and causes missorting of Chs3 to the vacuole [46]. Chs3 is responsible for the majority of chitin synthesis in the cell wall and the disruption of its normal cycling between the Golgi and the plasma membrane in the *SAC1* mutant compromises cell wall maintenance [46]. Deletion of *SAC1* substantially reduces strain fitness (to 0.48 relative to wild-type) and this is further aggravated by deletion of either *SCS3* or *YFT2*. However, the fitness of the triple mutant strain is significantly improved (to 0.78 relative to wild-type) indicating the strong alleviating effect of deleting both *SCS3* and *YFT2*. On the other hand, deletion of *CHS3* does not have a significant impact on strain fitness by itself but exhibits reduced fitness in combination with either *SCS3* or *YFT2*. These effects are suppressed in the triple mutant strain. The converse of this pattern of genetic interactions was also seen (albeit less often) where each query gene-deletion had alleviating interactions with a particular array gene deletion that was reversed when both *SCS3* and *YFT2* were deleted. These types of genetic interactions indicate that the functions of *SCS3* and *YFT2* (or the processes that they impact) sometimes antagonize one another.

### Functional enrichment of GO bioprocesses

**Figure 5:**
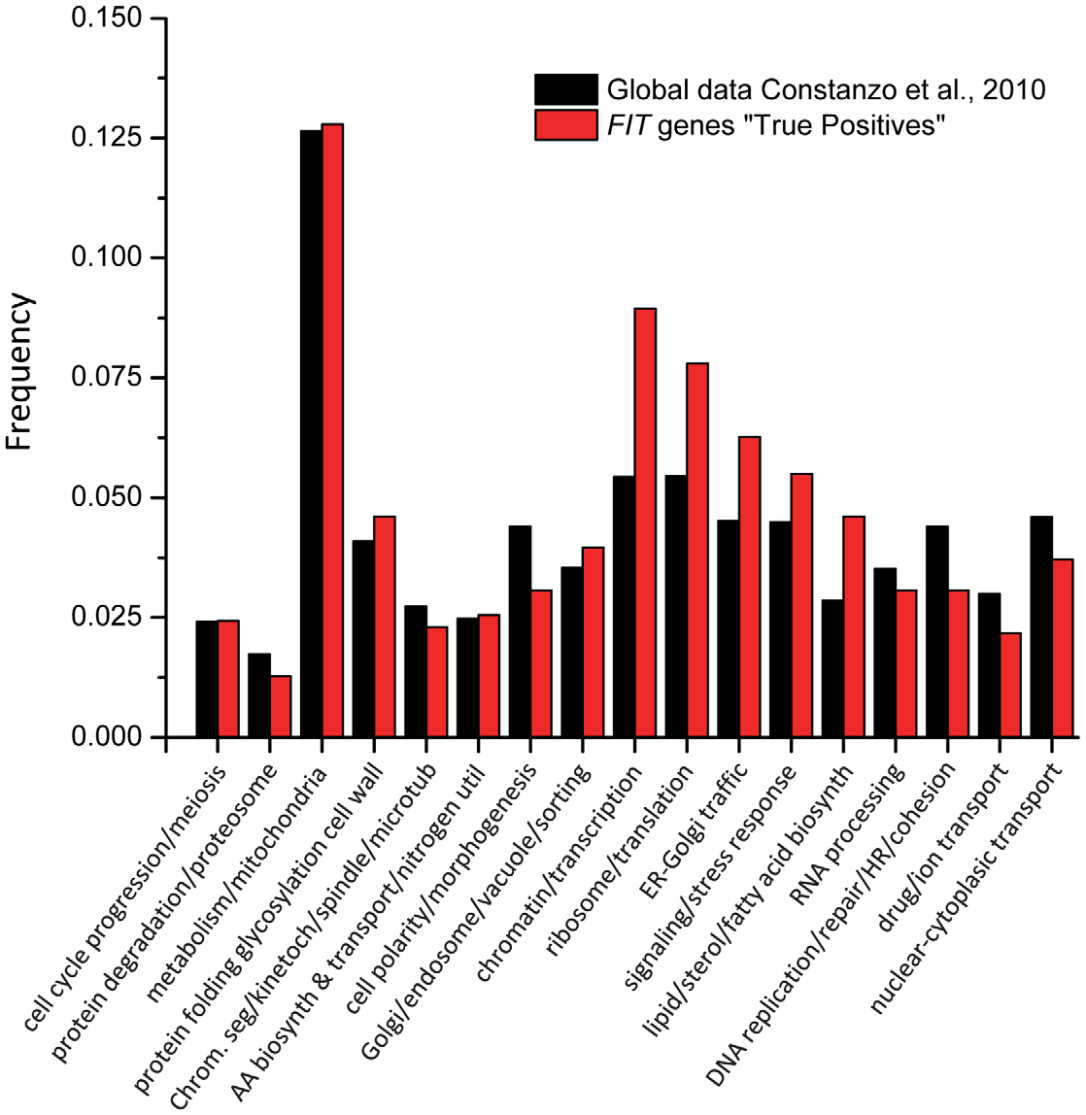
Enrichment of GO bioprocess terms among genes that have genetic interactions with *SCS3* and/or *YFT2*. The frequencies of 17 broad GO biological process terms [42] represented among the genes on the deletion array were compared with the 636 genes that interacted with *SCS3* and/or *YFT2*. Functional enrichment was calculated by hypergeometric distribution.

To identify the biological processes that are enriched among the genes identified in our screens, we compared the frequencies of 17 broad GO bioprocess terms among array genes [42] and the 636 genes that interacted with *SCS3* and/or *YFT2*. Five GO bioprocess terms were significantly over-represented in our data (Figure 5): Chromatin and transcription, ribosomes and translation, lipid, sterol and fatty acid biosynthesis, ER-Golgi traffic and signaling-stress response. These functional associations illustrate the fundamental importance and broad impact of *SCS3* and *YFT2* in the cell: They linked together processes (e.g. chromatin-transcription and secretion) that individually are among the most highly connected in the global genetic landscape [42]. This is further reflected by a yeast GO-slim component analysis which shows that the cellular distribution of genes that are synthetic with *SCS3* and/or *YFT2* is very similar to the genome as a whole (Spearman Rank Order Correlation r_s_ = 0.87 for the top 18 terms, even after excluding the cytoplasm and nucleus, which are the most abundant terms, Table S2). Consistent with the localization of the mammalian *FIT* proteins and Scs3 to the ER, this compartment was the most significantly overrepresented among genes interacting with *SCS3* and/or *YFT2* (p = 0.002) followed by the Golgi and the mitochondrial envelope (Table S2). Interestingly, a yeast GO-slim component analysis of genes that were synthetic with either *SCS3* or *YFT2* revealed an over-representation of the former with the ER and the latter with the plasma membrane and the mitochondrial envelope suggesting a cellular bias in their functional relationships.

### Genetic interactions and lipid metabolism

Given the effects of the mammalian *FIT* genes on LD formation and the enrichment of genetic interactions linking *SCS3* and *YFT2* to lipid metabolism (Figure 5, [10], [14]), we were interested to know whether the *scs3*Δ *yft2*Δ strain had altered levels of phospho- and/or neutral lipids. To assess this, we performed metabolic labeling of the wild-type and double deletion strains using either ^14^C-acetate or ^32^P-orthophosphate and analyzed the cell extracts by thin-layer chromatography. In parallel, we also prepared unlabeled samples for quantitative mass spectrometry. These analyses did not reveal any significant differences in the levels of total cellular neutral or polar lipid species (Figure S6 and data not shown).

**Figure 6:**
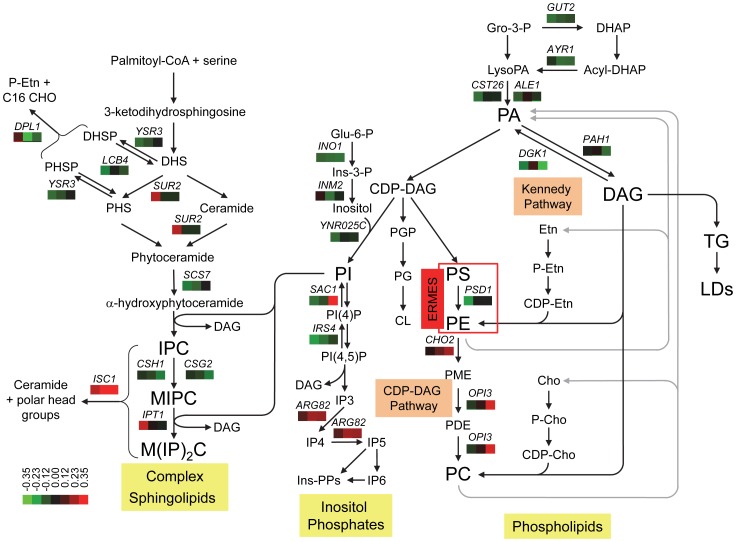
Genetic interactions of *SCS3* and *YFT2* map to sphingolipid, phospholipid, and inositol phosphate synthesis pathways. Cross-talk between the different pathways is illustrated by the central role of PI synthesis and DAG production/utilization. The arrangement of the steps in the Kennedy and CDP-DAG pathways for the synthesis of PC is modified from Carman and Han [47]. The role of the ERMES complex in transporting PS from the ER to the mitochondria for conversion to PE and its return to the ER [50] is represented by a red box. Genes encoding all four components of the complex (*MDM10, MDM12, MDM34 and MMM1*) exhibited alleviating interactions when both *SCS3* and *YFT2* were deleted (Table S1; the alleviating interaction between *MDM10* and the *SCS3 YFT2* double deletion was identified with relaxed criteria, 38 pixel size difference, *p* = 0.006, ε = 0.18). The synthesis of PE and PC from lysoPE and lyso PC is omitted for clarity. The steps in the synthesis of inositol phosphates are modified from York [52]. The pathway for the synthesis of complex sphingolipids is modified from Dickson [53]. Steps involving genes that interact genetically with *SCS3* and/or *YFT2* show the gene name above three boxes which indicate the relative strength and sign of the interactions. Aggravating (green) and alleviating (red) interactions are ordered from left to right for the *scs3* **Δ**, *yft2* **Δ** and *scs3* **Δ** *yft2* **Δ** strains using the values in Table S1. A colorbar shows the intensity of the interactions. Phosphatidic acid, PA; diacylglycerol, DAG; triglyceride, TG; lipid droplet, LD, Gro, glycerol; DHAP, dihydroxyacetone phosphate; Glu, glucose; Ins, inositol; PME, phosphatidyl monomethylethanolamine; PDE, phosphatidyl dimethylethanolamine; Etn, ethanolamine; Cho, choline., dihydrosphingosine, DHS; phytosphingosine, PHS; inositolphosphoceramide, IPC; mannosyl-inositolphosphoceramide, MIPC; mannosyl-diinositolphosphoceramide, M(IP)2.

To understand how the lipid metabolic functions identified in our screens affected strain fitness, we mapped the genetic interaction data onto known lipid metabolic pathways using gene descriptions annotated in SGD. Despite the fact that some of the genes in these pathways are essential or were otherwise absent from the deletion array, this analysis identified genetic interactions affecting multiple biochemical steps in the synthesis of phospholipids, inositol phosphates and sphingolipids (Figure 6). Additional interactions identified genes that function in sterol and fatty acid metabolism (Table S1). Mutations that negatively impact the synthesis of inositol or its conversion into PI showed aggravating interactions. This was also true for mutations that are defective in the synthesis of PA from glycerol-3-phosphate (Gro-3-P), DHAP and LysoPA (Figure 6). These effects are consistent with PA and the CDP-DAG pathway providing the main route for the synthesis of PI and the other major phospholipids (Figure 6) [47]. We infer that deletion of *SCS3* and/or *YFT2* impairs the synthesis of PI in a manner that is further exacerbated by deletion of different components in this pathway. In contrast to the synthesis of PI, deletions of the methyltransferases (*CHO2* and *OPI3*) that convert PE to PC in the terminal steps of the CDP-DAG pathway had the opposite phenotype (Figure 6). Deletions of both *SCS3* and *YFT2*, which together have little effect on fitness compared to the wild-type strain, suppressed the more severe fitness defect of the *cho2*Δ and *opi3*Δ strains. These alleviating phenotypes suggest that deletion of *SCS3* and *YFT2* may compensate for the reduced synthesis of PC in the methyltransferase mutant strains [48], [49]. Similarly, alleviating interactions were also found with deletions of all four components of the ERMES complex, an ER-mitochondrial tethering complex that is important for the efficient exchange of PS and PE between these compartments [50]. These interactions and the fact that alleviating phenotypes tend to be associated with genes in a common pathway or process [44], [45] predict that deletion of *SCS3* and *YFT2* can bypass defects in the CDP-DAG pathway related to PC synthesis.

A possible mechanism by which deletion of *SCS3* and *YFT2* could increase the fitness of the *cho2*Δ and *opi3*Δ strains involves increasing the flux of acyl chains and DAG through the Kennedy pathway for PC synthesis (Figure 6). In this way, DAG could be diverted from conversion into TGs, consistent with the reduction in LD formation upon knockdown [10] or loss of function of the mammalian *FIT* genes [14]. In support of this hypothesis, we found that deletion of the choline/ethanolamine transporter, Hnm1, resulted in aggravating genetic interactions in *scs3*Δ and *scs3*Δ *yft2*Δ strains indicating the importance of a functional Kennedy pathway in these strains (Table S1).

In addition to being a major cellular phospholipid, PI is also a key substrate in the synthesis of inositol phosphates and complex sphingolipids [46], [51]–[53]. Notably, deletion of genes in downstream steps in these pathways showed strong alleviating phenotypes with *SCS3* and *YFT2* (Figure 6). The turnover of complex sphingolipids, which comprise ∼12 mol percent of the yeast lipidome [51], is critical for survival in mammals and is important in yeast for signaling the response to different types of cellular stress [53]. This turnover reaction is catalyzed by Isc1, a phospholipase C-type enzyme that removes the polar head groups from complex sphingolipids to regenerate ceramides. Isc1 function confers resistance to a variety of cellular stresses including heat shock where ceramide levels rise dramatically to affect a transient arrest of the cell cycle and induce synthesis of the cryoprotectant trehalose [53]. Similarly, Isc1 is critical for growth on non-fermentable carbon sources and is known to change its localization from the ER to the outer leaflet of the mitochondrial membrane during the shift from fermentation to respiratory metabolism. Subsequent interactions with mitochondrial lipids such as cardiolipin increase Isc1 enzyme activity and thus the level of phytoceramide [53]. Accumulated evidence suggests that the ceramide generated by this turnover functions as a signaling molecule affecting numerous processes [53]. Accordingly, the ability of *SCS3* and *YFT2* gene deletions to rescue the defective growth of the *isc1*Δ strain may involve enhanced ceramide signaling or enhanced function of a ceramide-regulated process. We note that the underlying mechanism of this enhanced functionality is not likely to involve elevated de novo synthesis of ceramide or complex sphingolipids since deletions of multiple components in this pathway showed aggravating phenotypes (negative genetic interactions) in combination with deletions of *SCS3* and/or *YFT2* (Figure 6).

A role for *SCS3* and *YFT2* in cellular signaling is also suggested in relation to soluble inositol phosphates (IPs) since deletion of either one or both genes rescues the poor growth of a strain lacking Arg82, the inositol polyphosphate multikinase responsible for synthesizing IP4 and IP5 (Figure 6, [52]). Deletion of *ARG82* has pleiotropic effects on cellular function including gene transcription, nuclear mRNA export and telomere elongation. Current data suggest that these processes are regulated by IP4, IP5 and/or their more phosphorylated forms (IP6 and pyrophosphate derivatives) which function as controlling ligands for different biochemical activities (e.g. the ATP-dependent RNA helicase Dbp5, the INO80, SWI/SNF and RSC chromatin remodeling complexes and the Pho80-Pho85 cyclin-dependent kinase, [54]). Since Arg82 is the only enzyme known to synthesize IP4 and IP5 in yeast, it is not clear how deletion of *SCS3* and *YFT2* might bypass their absence in the *arg82*Δ strain. Other effects on inositol phosphorylation are suggested by genetic interactions with genes encoding several PI kinases and phosphatases or their regulators (e.g. *SAC1*, *IRS4*, *YMR1* and *FAB1*, Figure 6 and Table S1). Notably, deletion of the *Sac1* phosphatase, with its high selectivity towards PI(4)P (noted above), suppresses defects associated with a temperature-sensitive mutant of the opposing PI 4-kinase, *Stt4*, localized at the plasma membrane [46]. The specificity of this suppression, which is not seen for other PI-4- kinases, together with other evidence [46], supports the existence of discrete pools of PI(4)P with specific cellular functions and suggest a close physical association between the ER and the plasma membrane [46]. Based on this knowledge, suppression of the growth defect of the *sac1*Δ strain by deletion of both *SCS3* and *YFT2* could involve or impact functions at the plasma membrane.

The strong genetic associations that link *SCS3* and *YFT2* to the synthesis of phospholipids, sphingolipids and inositol phosphates are reflected in the cross-talk between these pathways involving lipid-derived second messengers such as phosphatidic acid (PA) and diacylglycerol (DAG) [47], [55], [56]. These associations are reinforced by genetic interactions involving *PAH1* and *DGK1* which encode PA phosphatase (lipin) and DAG kinase, respectively. Deletions of *PAH1* and *DGK1*, which catalyze the interconversion of PA and DAG [57], [58], resulted in aggravating genetic interactions with *SCS3* and *YFT2* (Figure 6) and suggest that balancing the levels of these lipid precursors/signaling molecules may be important for optimal growth. Proof of a signaling role for DAG in yeast via the canonical mechanism of PKC activation has proven elusive [55] but DAG generated by Pah1 has recently been implicated in LD biogenesis: Deletion of *PAH1* reduces the number of LDs and this effect is suppressed if the cells are also deleted for *DGK1* [59]. Given the ability of the mammalian *FIT* genes to bind DAG and TG and their associated LD phenotypes [10], [14], the genetic interaction between *SCS3/YFT2* and *PAH1* is entirely consistent with a functional association relating DAG metabolism and LDs. In addition, deletion of *PAH1* (or overexpression of Dgk1) elevates the concentration of PA which up-regulates the transcription of phospholipid biosynthetic genes and drives a dramatic expansion of the nuclear/ER membrane (reviewed in [60]). This is achieved by controlling the nuclear concentration of the transcriptional repressor Opi1 which is otherwise sequestered on the ER membrane in a complex with PA and the tail-anchored protein Scs2 [60]. Genetic interactions with this machinery are presented in the next section. Overall the interactions described above provide further evidence that *SCS3* and *YFT2* function in yeast affects lipid signaling and homeostasis.

### Genetic interactions and transcription

**Figure 7:**
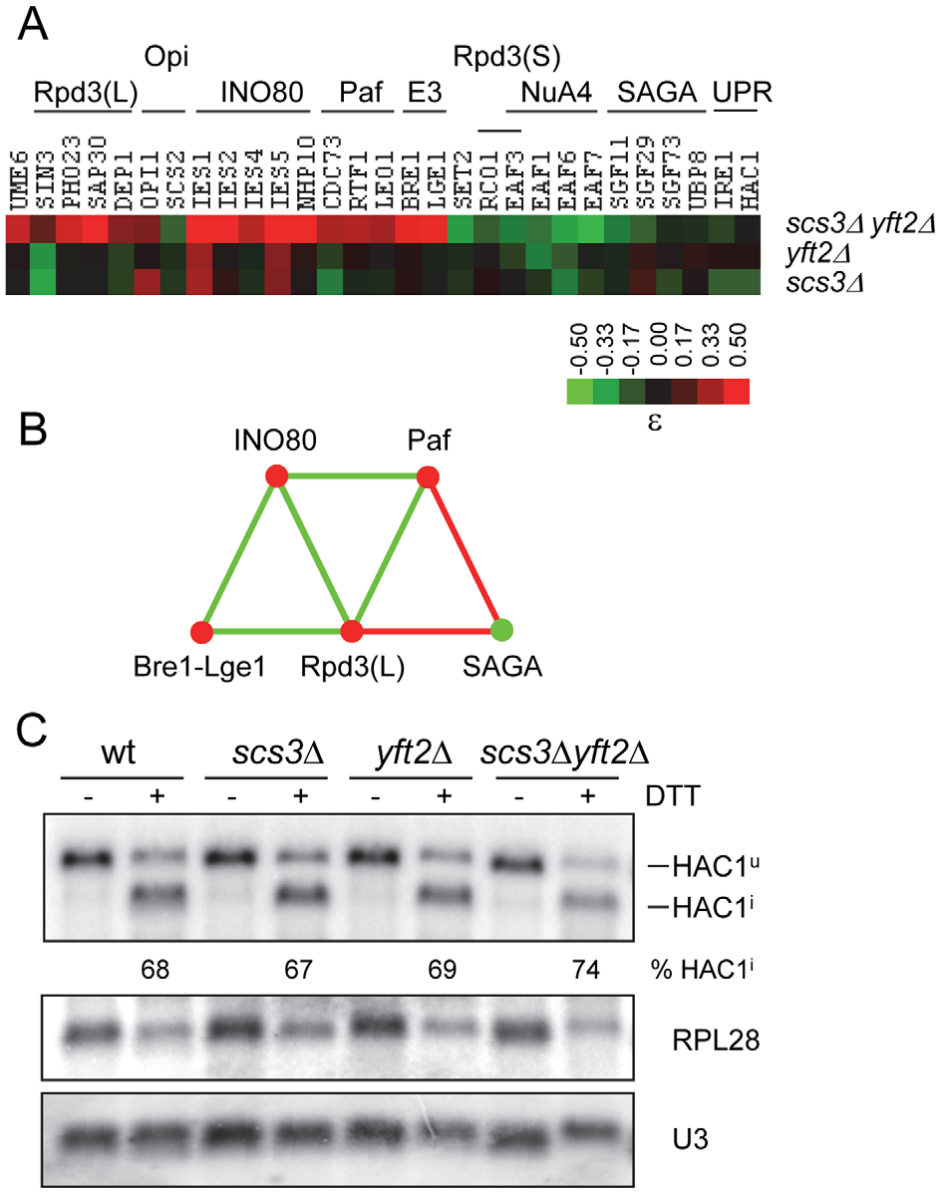
Functional relationships between *SCS3* and *YFT2* and transcriptional components associated with the expression of phospholipid biosynthetic genes. (A) The heat map shows array genes that interact genetically with deletions of *SCS3*, *YFT2* or both genes. The strength of the interactions is indicated by the colorbar. Array genes are grouped according to the transcription complexes or activities/pathways that define their function as indicated above the heat map. Supporting interactions that are not represented in the high stringency set were identified using relaxed criteria and include *UME6* and *LGE1* (p = 0.02), *DEP1*, which had a 39 pixel size difference just below the 40 pixel cutoff and *UBP8* (ε = −0.09 in the triple mutant). Genetic interactions with *EAF1* were identified with *OPI7* which encodes a dubious ORF that overlaps the N-terminus and promoter of *EAF1*. (B) Genetic relationships between transcription components that interact with *SCS3* and *YFT2* (panel A) were determined by searching the DryGin database [78]. This resulted in the identification of three linked triplet genetic motifs. Each edge connecting the complexes in this network represents a minimum of three interactions, either all alleviating (red) or all aggravating (green). The same color scheme is used for nodes which indicate the type of interaction of the different complexes with the *SCS3 YFT2* double deletion strain (as in panel A). (C) Analysis of the unfolded protein response (UPR) pathway in *SCS3* and *YFT2* gene-deletion strains. Northern analysis of *HAC1* mRNA (reporting both unspliced Hac1^u^ and spliced Hac1^i^ forms), stable U3 snRNA and *RPL28* mRNA was performed on RNA from early log phase cultures (SC-medium) of wild-type and double deletion strains before and after DTT treatment. The Hac1 band intensities were used to calculate the distribution between spliced and unspliced forms. Normalized *HAC1* mRNA band intensities were used to determine the level of transcription relative to the wild-type untreated strain. The *RPL28* mRNA levels are included to report the global cellular stress response to DTT treatment. Results from duplicate independent samples of wild-type and double deletion strains are reported under the representative autoradiogram.

Overexpression of Nte1, a phosphatidylcholine B-type phospholipase, suppresses the inositol auxotrophy of *scs3*Δ (and several other Ino- strains) and restores normal levels of Ino1 protein [37]. Similarly, the Ino- phenotype of a large number of gene-deletion strains, including *SCS3*, can be suppressed by deleting the Opi1 repressor [61] which leads to constitutive expression of UAS_INO_-regulated genes, increased inositol synthesis and altered lipid composition [31], [62], [63]. These data suggest that the inositol auxotrophy of *scs3*Δ strains (Figure 1) could be due to misregulated *INO1* gene transcription. Consistent with this, we found that gene-deletions yielding synthetic phenotypes with *SCS3* and *YFT2* were enriched for known transcription components (Figure 5) and many of these interactions involve regulators of UAS_INO_ genes [32]. For example, deletions of known transcriptional repressors were found to have alleviating phenotypes when combined with deletions of both *SCS3* and *YFT2* (Figure 7). Among these were four subunits of the Rpd3(L) histone deacetylase (HDAC) complex (*SIN3*, *SAP30*, *PHO23* and *DEP1*) which is recruited to the *INO1* promoter via its interaction with the DNA binding factor Ume6 [32]. The fitness of these deletion strains is improved by deleting both *SCS3* and *YFT2* and this presumably coincides with reduced transcription of *INO1* and other target genes in the triple mutants relative to the Rpd3(L) single mutants (Figure 7). Conversely, deletions of activators or anti-repressors of UAS_INO_-regulated genes were found to have aggravating phenotypes in the *scs3*Δ *yft2*Δ strain (Figure 7). Notably, deletion of *SCS2* which tethers Opi1 to the ER, and subunits of the SAGA chromatin modifying complex, resulted in synthetic fitness defects, consistent with *SCS3* and *YFT2* gene deletions reducing the already diminished transcription of UAS_INO_-regulated genes in these strains. Interestingly, the SAGA subunits are linked genetically and biochemically to other components of the transcription machinery that also interact with *SCS3* and *YFT2* [64], [65]. Deletions of the genes encoding these transcription components reveal a coherent pattern of genetic interactions that is consistent with current models of their function in transcription (Figure 7a–7b): Alleviating interactions were found for the Bre1-Lge1 E3 ubiquitin ligase complex along with several subunits of the Paf1 complex which together are important for monoubiquitination of histone H2B during transcription initiation and elongation (Figure 7, [64]). In contrast, the failure to deubiquitinate H2B has negative consequences for cellular fitness in the *scs3*Δ *yft2*Δ strain based on the aggravating phenotypes of SAGA subunit deletions in its deubiquitination module (Sgf11 and Ubp8) and in Sgf73 which tethers this module to the rest of the SAGA complex [65]. This is consistent with the important role of H2B deubiqutination for the recruitment of kinases, such as Ctk1, to the elongating polymerase [64], [65]. Subsequent phosphorylation of serine 2 in the carboxy terminal domain of RNA polymerase II provides a binding site for the Set2 methyltransferase leading to histone H3 K36 trimethylation. Recent work has shown that this modification is important for activated transcription of UAS_INO_-regulated genes [66] and for recruitment of the Rpd3(S) HDAC complex which inhibits cryptic initiation within the transcription unit [67]. Accordingly, deletions of *SET2* and specific subunits of the Rpd3(S) complex (*RCO1* and *EAF3*) exhibit aggravating interactions with *SCS3* and *YFT2* (Figure 7a).

Other aggravating interactions were identified with subunits of the NuA4 lysine acetyl transferase complex which acetylates histone H2A and its variant H2A.Z along with histone H4 (Figure 7). Like SAGA, NuA4 is recruited to the promoters and coding regions of genes and cooperates with SAGA in nucleosome disassembly and transcription elongation [68]. Although NuA4 has not been physically associated with UAS_INO_ genes, NuA4 mutants are inositol excretors implying a paradoxical increase in *INO1* transcription [69]. The mechanism underlying this phenotype is unknown.

Induction of *INO1* transcription upon inositol starvation involves the repositioning of nucleosomes in the promoter by the INO80 chromatin remodeling complex [70]. In contrast to other activators of UAS_INO_ gene transcription (e.g. SAGA), deletions of multiple INO80 subunits led to alleviating rather than aggravating phenotypes in the *scs3*Δ *yft2*Δ strain (Figure 7). This apparent inconsistency is explained by the functional redundancy of INO80 with another chromatin remodeler, SWI/SNF, which masks the effect of INO80 subunit deletions on *INO1* gene activation [71]. Additionally, the INO80 complex serves other functions in the cell [70], [72], [73]. These include the reassembly of nucleosomes in the transcribed regions of genes to repress transcription during adaptation to cellular stress [72]. Thus, our observations are consistent with the loss of *SCS3* and *YFT2* rescuing the poor fitness of INO80 complex mutants that are deficient in a repressive function in transcription. This interpretation is supported by strong negative genetic interactions between the INO80 complex and the Rpd3(L) HDAC complex indicating that they function in redundant parallel pathways (Figure 7, [42], [44]).

*INO1* transcription is also positively regulated by activators of the unfolded protein response (UPR) pathway, namely the Ire1 sensor kinase/endoribonuclease and the Hac1 transcription factor [74]. Maximal levels of *INO1* gene transcription requires Ire1 and Hac1 and starvation for inositol (reviewed in [32]). Conversely, deletion of *IRE1* or *HAC1* confers inositol auxotrophy [75], [76]. Consistent with these observations, we found that *IRE1* and *HAC1* exhibited aggravating genetic interactions with *SCS3* and *YFT2*, similar to other positive regulators in this system (Figure 7, see above). Although activation of the UPR by heat stress does not induce *INO1* transcription [32], the role of *IRE1* and *HAC1* in this process has led to the view that inositol starvation and the resulting changes in lipid metabolism and stress response pathways causes stress on the ER (e.g. see refs. [37], [60]). Given the known localization of Scs3 and mammalian FIT proteins in the ER membrane [10], [77] and the negative genetic interactions with *IRE1* and *HAC1*, we examined whether deletion of *SCS3* and *YFT2* creates an ER stress that constitutively activates the UPR. For cells grown in synthetic complete medium, northern analysis of *HAC1* mRNA splicing revealed no evidence for UPR induction in the *scs3*Δ *yft2*Δ strain and a normal UPR response to acute treatment with DTT (Figure 7c). In summary, the data presented in this section indicate that deletion of *SCS3* and *YFT2* perturbs the regulation of phospholipid biosynthetic genes and sensitizes the cell to additional changes in transcriptional activities that are also involved in this process.

### Genetic interactions and protein synthesis

**Figure 8:**
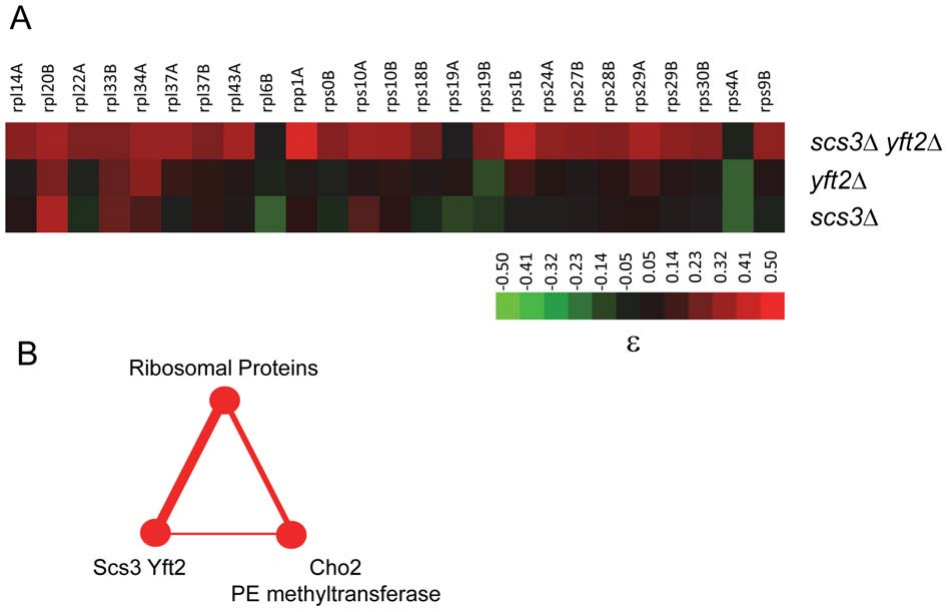
Genetic interactions of *SCS3* and *YFT2* linking protein and phospholipid synthesis. (A) The heat map shows ribosomal protein genes that interact genetically with deletions of *SCS3*, *YFT2* or both genes (data were extracted from Table S1). The strength of the interactions is indicated by the colorbar. (B) Triplet genetic motif representing mutually alleviating interactions (red nodes and edges) between multiple RP genes, *SCS3*/*YFT2* and *CHO2*. The thickness of the edges reflects the number of interactions. They include 25 alleviating or suppressing interactions between RP genes and *SCS3/YFT2* (from panel A), five of which were also alleviating with *CHO2* (i.e. *RPS29B*, *RPS18B*, *RPS1B*, *RPL43A* and *RPS19B* [42]) and the interaction of *SCS3/YFT2* with *CHO2* (see Figure 6).

Among the genetic interactions affecting protein synthesis, we found that reduced growth due to pseudo-haploinsufficiency of multiple RPs was suppressed by deletion of both *SCS3* and *YFT2* (Figure 8). This phenotype was not restricted to RPs but was also seen for positive regulators of ribosome biogenesis such as the transcription factor *HMO1* and proteins involved in rRNA processing (e.g. Bud21) and ribosome assembly (e.g. Rei1) (Table S1). These results imply that the characteristic reduced growth of RP gene-deletion strains is not simply due to a reduction in the number of ribosomes but includes a fitness defect (i.e. a biological imbalance with other processes) that can be corrected upon deletion of both *SCS3* and *YFT2*. To gain some insight into the nature of these processes, we examined all of the alleviating interactions identified in screens of RP gene-deletion strains reported by Costanzo et al [42], [78]. With the exception of three RP genes, the most frequently recovered alleviating gene-deletion (in 10 of 37 screens) was the Cho2 methyltransferase involved in the conversion of PE to PC. Importantly, *CHO2* also exhibits alleviating interactions with *SCS3* and *YFT2* (Figure 6). These interactions include five triplet genetic motifs involving mutually positive interactions (i.e. five different RP genes interacting with both *CHO2* and *SCS3/YFT2*, Figure 8). Together with numerous additional interactions between RP genes and either *CHO2* or *SCS3/YFT2*, the data strongly suggest that these genes are associated via a common pathway [79]. We propose that the functional significance of these associations is to balance the energy-intensive synthesis of ribosomes with the synthesis of phospholipids in the ER to achieve optimal strain fitness for both of these growth-related processes. In support of this view, it has been known for some time that defects in the secretory pathway that block the delivery of vesicles to the plasma membrane (and thus inhibit plasma membrane expansion) activate a Rho1-Pkc1-dependent signaling pathway to repress transcription of rRNA, tRNA and RP genes [80]–[82].

### A role for *SCS3 and YFT2* in specific ER stress responses

**Figure 9:**
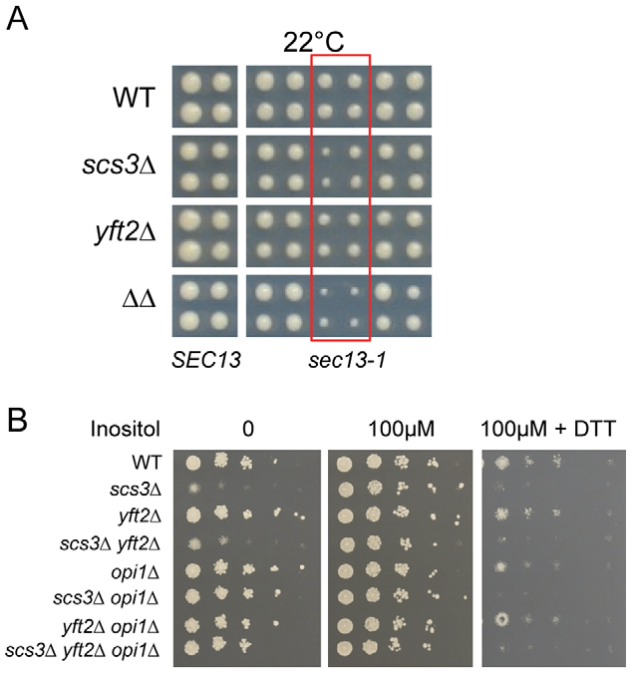
Hypersensitivity of *SCS3* and *YFT2* deletion strains to secretory/ER stress. (A) Wild-type and gene-deletion strains were crossed to an array of temperature-sensitive strains (gift from Charlie Boone) and haploid single, double or triple mutant strains generated by SGA methods were printed at the permissive temperature of 22°C. Images from the final selection plates show quadruplicate colonies for each array strain including a wild-type *SEC13* control strain (left panels). Strains containing the *sec13-1* mutation are boxed in red and are flanked on either side by other temperature-sensitive strains that are not synthetic with *SCS3* or *YFT2*. (B) Wild-type and gene-deletion strains were spotted onto SC media with no inositol, 100 µM inositol, and 100 µM inositol plus 2.5 mM DTT. Plates were photographed at 2 days.

Deletion of *SCS3* and *YFT2* resulted in strong aggravating phenotypes with deletions such as *SEY1* and *ICE2* (proteins linked to ER morphology) and components of the secretory pathway (Table S1) [83]. This suggested a requirement for *SCS3* and *YFT2* in normal ER function. To examine this relationship further, we followed the kinetics of vacuolar carboxypeptidase Y processing and maturation as an indicator of protein trafficking between the ER, the Golgi and the vacuole. No gross defects were evident in the *scs3*Δ *yft2*Δ strain (Figure S7a). In a more stringent test of ER function, we employed a conditional allele of *SEC13* (*sec13-1*) that causes secretory stress and activates the UPR as a result of defects in COPII-mediated vesicle formation [84]. Deletion of *YFT2*, *SCS3* or both genes in the *sec13-1* strain caused an increasingly strong synthetic sick growth phenotype at the permissive temperature of 22°C (Figure 9a) and at higher temperatures (data not shown). The *sec13-1* strain exhibits altered lipid metabolism at 25°C and upon shifting to its non-permissive temperature, accumulates TGs at the expense of phospholipid synthesis with a concomitant increase in the number of LDs [85]. However, deletion of *SCS3* and *YFT2* did not affect the number, apparent size or distribution of LDs in this strain despite the synthetic growth defect (in either log phase, Figure S3c or stationary phase, data not shown). Thus, the strong aggravating phenotype of the *sec13-1 scs3*Δ *yft2*Δ strain may be explained by hypersensitivity to secretory stress alone or in combination with altered phospholipid composition. Indeed, deletion of *SCS3* has been reported to cause elevated UPRE-reporter gene expression [86], [87] and to confer hypersensitivity to tunicamycin in UPR-defective strains, consistent with elevated levels of ER stress [88]. These observations prompted a closer evaluation of *SCS3* and *YFT2* function during induction and attenuation of the UPR.

**Figure 10:**
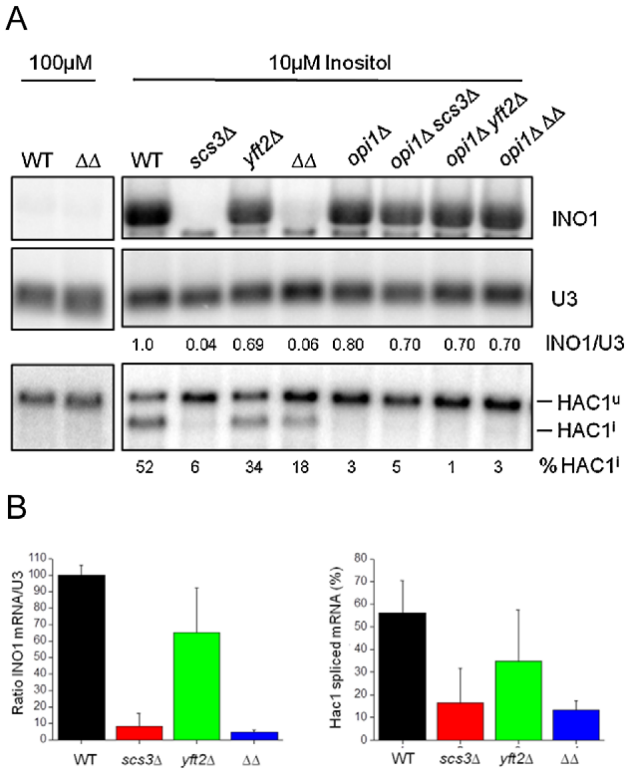
*SCS3* and *YFT2* gene-deletion strains are defective in the transcriptional response to low inositol. (A) Northern analysis of wild-type and gene-deletion strains grown in low inositol media. Cells were grown overnight to early log phase at 30°C in the presence of 10 µM inositol before RNA preparation. RNA from the same strains grown in 100 µM inositol is included for comparison. Detection and quantitation of *INO1* and *HAC1* mRNAs and U3 snRNA was as described in Figure 7c. The amount of *INO1* mRNA normalized for U3 snRNA is expressed relative to the wild-type strain as indicated under each lane. The extent of Hac1 splicing is expressed as % of total *HAC1* mRNA, normalized for U3 snRNA and is indicated under each lane. Note that deletion of *OPI1* results in constitutive *INO1* transcription and inositol prototrophy for the *SCS3* and *YFT2* deletion strains (see also Figure 9b), thereby alleviating the UPR response to low inositol (i.e. no Hac1 splicing in *opi1*Δ strains). (B) Data from three independent low inositol experiments, as shown above, are plotted ± standard deviation. The left panel shows the relative level of Ino1 mRNA and the right panel the extent of *HAC1* mRNA splicing for each strain.

Strains deleted for *SCS3* and *YFT2* are indistinguishable from wild-type in their acute response to DTT-induced ER stress in nutrient-replete medium (i.e. normal *HAC1* mRNA splicing as described above, Figure 7c) and do not exhibit significant sensitivity to tunicamycin (Figure S7b). In contrast, *scs3*Δ strains are hypersensitive to growth on medium containing DTT (Figure 9b) suggesting an inability to tolerate the accumulation of unfolded proteins and implying a role in resistance to chronic ER stress. To further explore this possibility, we examined growth in inositol-depleted medium, a UPR-inducing condition that is independent of unfolded protein accumulation [87]. In wild-type strains, low concentrations of inositol (e.g. 10 µM, sufficient to rescue the inositol auxotrophy of *scs3*Δ strains) induce transcription of phospholipid biosynthetic genes (e.g. *INO1*) and activate the UPR (increase *HAC1* pre-mRNA splicing) [31] (Figure 10, compare 10 µM versus 100 µM inositol). However, the *SCS3* and *YFT2* deletion strains were significantly compromised for both of these responses (Figure 10). Importantly, deletion of the Opi1 transcriptional repressor (which confers inositol prototropy to *scs3*Δ strains and alters the expression of many UAS_INO_ genes [31], [61]), restored wild-type levels of *INO1* transcription to the *SCS3* and *YFT2* deletion strains (Figure 10). That *SCS3* was identified along with two known regulators of UAS_INO_ gene transcription (*SCS2*, [89] and *SCS1/INO2*, [90]) in a screen for suppressors of a choline-sensitive mutant [36], coupled with its inability to derepress *INO1* transcription (Figure 10), suggests that the primary defect underlying the inositol auxotrophy of *scs3*Δ strains is the failure to appropriately regulate transcription of *INO1* and other genes involved in lipid metabolism.

Phospholipid biosynthesis is induced as part of the normal response to ER stress [91], yet a failure to inactivate Ire1 and attenuate *HAC1* mRNA splicing and the UPR has recently been reported to reduce cell survival under conditions of chronic ER stress [92], [93]. We found that the hypersensitivity of *SCS3* deletion strains to growth on medium containing DTT was independent of the transcriptional repressor Opi1 (Figure 9B), suggesting that the constitutive lipid biogenesis and membrane expansion that follows deletion of *OPI1* [91] is not sufficient to rescue the ER stress-induced growth defect in this strain. Indeed, attenuation of the UPR, as detected in a time course of *HAC1* mRNA splicing after washout of DTT was compromised in *SCS3* deletion strains (Figure S8, and data not shown). Together, the preceding data suggest a requirement for *SCS3* and *YFT2* in the normal response to and recovery from ER stress under specific conditions: Chronic exposure to unfolded protein accumulation induced by DTT and growth in low inositol. The defective transcriptional response of *scs3*Δ and *yft2*Δ strains to low inositol (Figure 10) suggests that Scs3 and Yft2 may be required for phospholipid biosynthesis in response to ER stress, perhaps contributing to the retention of Opi1 at the ER.

### Conclusion

The genetic interactions of *SCS3* and *YFT2* reveal functional relationships with a large network of genes and with biological processes that are among the most highly connected in the overall genetic landscape of the cell [42]. This high connectivity may reflect an important function of the corresponding proteins related to their potential binding of neutral lipids, their localization within the ER membrane and the role of the ER in secretion, stress response and transcriptional control. The finding that *SCS3* and *YFT2* deletion strains are compromised in the regulation of *INO1* transcription and fail to induce splicing of *HAC1* mRNA in response to growth in low inositol (Figure 10) demonstrates that these strains have an intrinsic defect in ER membrane function. Together the results suggest that *SCS3* and *YFT2* are required for normal ER membrane biosynthesis in response to perturbations in lipid metabolism and ER stress. The mechanisms linking Scs3 and Yft2 protein function to downstream cellular responses are unknown. However, the recent demonstration that the mammalian FIT proteins bind DAG and TG suggests that these interactions may affect local properties of the ER membrane such as its curvature or stability (see ref. [59]) or the local distribution of PA, altering the dynamics of Opi1-membrane interactions and the function of ER transmembrane proteins such as Ire1.

## Materials and Methods

### Yeast strains, media preparation, and plate assays

Synthetic complete media contained 400 µM myo-inositol unless otherwise stated. Low or no inositol media was prepared from inositol-free YNB (Difco) and amino acid dropout mixes. YPO media contained 0.3% yeast extract, 0.5% peptone, 0.1% glucose, 0.5% KH_2_PO_4_ and 0.1% oleic acid (YPO) and 0.2% Tween 80 [94]. For experiments other than SGA analysis, single, double and triple gene-deletions of *SCS3*, *YFT2* and *OPI1* were generated de novo in BY4742 using standard one step gene replacement and dominant drug selectable markers [95]. Paromomycin, DTT, cerulenin, oleate and fenpropimorph were purchased from Sigma-Aldrich.

### SGA analysis

Query strains were derived from Y7092 [96] by deleting *SCS3* (*scs3*Δ*::natMX*), *YFT2* (*yft2*Δ*::natMX*) or both genes (*scs3*Δ*::natMX yft2*Δ*::URA3*) and screened by standard SGA methods against an array of 4292 strains representing a healthy subset (i.e. minimal growth defects) of the viable gene-deletion collection [43], [96]. The gene-deletion array contained duplicate copies of each strain in a 1536 colony per plate format and was screened in triplicate for each query using a Singer Instruments RoToR hda robot. Digital images of the double or triple mutant colonies were captured and analyzed using ColonyImager software [43] to determine colony pixel sizes. These data were compared to colony pixel size data obtained from quintuplicate screens of Y8835 [96], a control query strain (*ura3*Δ*::natMX*), against the same deletion array.

### Data normalization and quantitation of genetic interaction strength

Each plate of the deletion array contained a two-colony perimeter of the same *his3*Δ*::kanMX* strain [96]. The 148 colonies on the inner perimeter compete for nutrients with their neighbors in a similar way as colonies located within this perimeter. These colonies provided a reliable measure for the normalization of colony size across all plates in the five control and nine query screens. Normalization followed the general scheme described by Collins et al., [97]. The median pixel size (area) of the *his3*Δ*::kanMX* colonies on the inner perimeter of each plate (the plate median) and of all 98 plates (experimental median, 343 pixels) was determined and used to normalize the colony sizes on each plate as follows: Normalized size = unnormalized size×343/plate median. Following normalization, the mean colony size (± standard deviation) was calculated for each mutant (represented by 10 colonies in the control screens and six colonies in each query screen) and the data were filtered to remove outliers (>2 standard deviations from each mean). After recalculation of the mean and standard deviation for each colony, the difference in the pixel size of each mutant on the array was computed between the control and query screens. The significance of this difference was assessed by performing a two-tailed t-test (with unequal variance) of the null hypothesis that the sizes of the colonies in the control and query screens are statistical the same. The fitness of the query strains relative to wild-type was determined by comparing the median *his3*Δ*::kanMX* colony sizes on the inner perimeter for all plates in each query screen versus the control screen (i.e. 3108 colonies for each query and 5180 colonies for the control). The resulting relative fitness values (W*scs3*Δ = 0.95, W*yft2*Δ = 0.96 and W*scs3*Δ *yft2*Δ = 0.89) were used to calculate genetic interaction scores (ε) as described below. The fitness of each array deletion strain as a single mutant or as a double or triple mutant (combining deletions of *SCS3* or *YFT2* or both genes) relative to wild-type was determined from the ratio of the mean colony size of the mutant to the median *his3*Δ*::kanMX* colony size from the 5180 control colonies. Genetic interaction scores for digenic pairs were calculated as the difference between the observed and expected fitness values of the double mutants where expected fitness was calculated as the product of the fitness of the corresponding single mutants (i.e. a multiplicative model, [41], [42]). Thus, ε = Wxy – (Wx * Wy) where Wxy is the observed fitness of the double mutant, Wx is the observed fitness of the array deletion strain and Wy is W*scs3*Δ or W*yft2*Δ (see above). For trigenic interactions, a similar multiplicative model was employed, ε = Wxyz – (Wx * Wyz), where Wxyz is the observed fitness of the triple mutant, Wx is the fitness of the array deletion strain and Wyz is W*scs3*Δ *yft2*Δ (see above). The general applicability of this solution in the current study is satisfied by the modest 11% fitness defect (W*scs3*Δ *yft2*Δ = 0.89) of the *scs3*Δ *yft2*Δ query strain relative to wild type. Unless otherwise indicated, subsequent analyses employed stringent criteria to select interactions with a high probability of representing true positives. These criteria included (i) ≥40 pixel size difference between single mutant and double or triple mutant colonies; (ii) a p value ≤0.01 representing the probability that the colony sizes of the strains being compared are significantly different from one another and (iii) ε scores ≤−0.12 or ≥0.2. Similar criteria for high stringency cutoffs were applied in the large scale study of Costanzo et al [42]. Selection of cutoffs for ε were also based on pairwise comparisons of 221 genetic interactions identified with two or more query strains (Figure 3b–3c, Figure S4). After removal of genes annotated as dubious ORFs in SGD, this analysis yielded a set of genetic interactions with 636 unique genes (Table S1). In addition to the metrics used for validating our screens we also compared our data to published SGA studies where genetic interactions have been reported for *SCS3*. In an E-MAP of the early secretory pathway [83], 29 aggravating genetic interactions with *SCS3* were reported that had an S score <−1.5. We scored 22 of these and found 14 (64%) that met our stringent criteria (pixel size difference, *p* value and ε). In addition, many genetic interactions with *SCS3* were identified by Constanzo et al., [42]. We downloaded these data from DRYGIN using the default values for SGA score and *p* value (IεI>0.08 and *p*<0.05). From this group we determined that 140 unique interactions were scored in our dataset. Forty-eight of these met our stringent criteria and a total of 64 interactions (46%) were found when interactions were compared at the same *p* value limit (*p*<0.05).

### GO bioprocess enrichment

Biological process annotations for genes on the viable gene-deletion array were obtained from supplementary data file S6 of Costanzo et al [42]. We calculated the frequencies of GO terms in this list and among the 636 genes that interacted with *SCS3* and/or *YFT2* and then calculated enrichment by hypergeometric distribution.

### Bioscreen growth curves

Growth at 30°C with shaking was measured at using a Bioscreen C Microbiology Reader (Growth Curves USA), which recorded OD_600_ readings from 100-well plates every 15 minutes. A single colony was inoculated at a starting OD_600_ of 0.1 into SD/MSG media that contained 0.1% Nonidet-P40. Doubling time and growth curve data were derived from three independent colonies per strain or condition as per established protocols [98], [99].

### Fluorescence microscopy

HEK293 cells were imaged as described in [14] *SI Materials and Methods*: “Microscopy” section. For yeast imaging an upright Olympus BX61 microscope with a sensicam QE cooled CCD camera (Black/White) and IP Lab 4.0.8 software was used at the Analytical Imaging Facility at the Albert Einstein College of Medicine. Cells were viewed with a 100X NA = 1.4 oil objective and FITC filter. Sections (0.2 µm) were collected for each image and combined into a single projection using Image J (http://imagej.nih.gov/ij/). Cells, either early log phase (between OD_600 nm_ 0.2 and 0.8), freshly saturated overnight or 5 day stationary phase cultures, were stained with 0.5 µg/µl BODIPY 493/503 (Invitrogen), for 5 to 10 min. prior to fluorescence microscopy. Cells were prepared as indicated from either SD/MSG, YPD, YPO or SC-Ino media and either imaged immediately or fixed in paraformaldehyde and permeablized in 0.1% Triton as described in [85] prior to staining and imaging.

### RNA isolation and northern blotting

Methods for RNA preparation and Northern analysis have been reported elsewhere [100], [101]. Yeast were grown to early log phase before harvesting or treatment and RNA extracted using the hot phenol method. End-labeled oligonucleotides specific for detection of Ino1, Hac1 and Rpl28 mRNAs and U3 snRNA were hybridized, detected by phosphorimage and analysed using ImageQuant software as described previously [100].

### In vivo labeling, isolation, and analysis of lipids

Metabolic labeling of lipids in log phase wild type and *SCS3* and *YFT2* deletion strains was achieved by growing strains for 2 hours in 1 µCi/ml of [1-^14^C]acetate (57 mCi/mmol) or for 6–8 generations in 10 µCi/ml ^32^P-orthophosphate. Lipids were extracted by the two-step 4°C method [51]. Sequential extracts in chloroform/methanol (17∶1, v/v) for 120 min and chloroform/methanol (2∶1, v/v) for 120 min were pooled, evaporated under N_2_ and dissolved in chloroform/methanol (1∶2, v/v) for thin layer chromatography. Labeled neutral and phospholipids were separated by one-dimensional TLC in hexanes∶ethyl ether∶acetic acid (80∶20∶1) [102] or chloroform∶ethanol∶triethanolamine∶H_2_0 (30∶35∶35∶7) [103], respectively. Plates were detected by phosphorimage and analysed with ImageQuant software. Extracts from 50 OD_600_ of log phase wild-type and *SCS3* and *YFT2* gene-deletion cells were prepared in triplicate and analyzed by mass spectrometry. Total lipid species are reported as nmol/10^8^ cells. Mass spectrometry was performed at the Kansas Lipidomics Research Center Analytical Laboratory.

## Supporting Information

Figure S1 Phylogenetic distribution of *SCS3* and *YFT2* in the genomes of sequenced fungi. Evolutionary relationships were constructed using the sequences of 153 genes that are universally present in the 42 genomes shown [26]. Major clades are indicated, including the *Saccharomyces* complex, the group of species that share the whole-genome duplication (WGD) and those with the variant genetic code (CTG). *YFT2* and *SCS3* orthologs were identified by Blast searches of yeast genomes at NCBI or the Broad Institute databases using the complete amino acid sequences for *SCS3* or *YFT2* or the corresponding signature sequences from the fourth transmembrane domain of each protein. *FIT* gene orthologs were identified in all organisms except *P. chrysosporium*, *A. terreus* and *C. globosum*. Figure was adapted from ref. [26].
(TIF)

Figure S2 Expression of HsFit2 in *SCS3 YFT2* gene-deletion strains. A HsFit expression complements synthetic growth defects in *scs3*Δ *yft*2Δ *xxx*Δ strains. The HsFit2 gene, controlled by a *TDH3* promoter and a *CYC1* terminator, was substituted for *YFT2* by one-step gene replacement in the Y7092 *scs3*Δ::*URA3* query strain. SGA techniques were then used to recover wild-type, *scs3*Δ *yft*2Δ and HsFit2-expressing *scs3*Δ *yft*2Δ strains containing the indicated deletions from the viable gene-deletion array. Final haploid colonies generated in quadruplicate were printed onto SGA selection media and photographed at 48 hours. Each row of panels is deleted for a different array gene (indicated on the right). Wild-type (WT; *SCS3 YFT2*), *scs3*Δ *yft*2Δ (ΔΔ) and HsFit2-expressing *scs3*Δ *yft*2Δ (ΔΔ HsFit2) strains are annotated across the top. Note that only the *ice2*Δ strain is an inositol auxotroph and the growth phenotypes were assayed on SGA medium which contains excess inositol. B HsFit expression in the *scs3*Δ *yft*2Δ strain does not induce the UPR. Northern analysis of the distribution of *HAC1* mRNA into unspliced Hac1^u^ and spliced Hac1^i^ forms and stable U3 snRNA was performed on RNA from early log phase cultures of wild-type, *scs3*Δ *yft*2Δ (ΔΔ) and HsFit2-expressing *scs3*Δ *yft*2Δ (ΔΔ HsFit2) strains grown in the presence of excess inositol. The extent of Hac1 splicing is expressed as % of total Hac1 mRNA and is indicated under each lane. ER stress in untreated and DTT-treated cells is reported by the accumulation of spliced *HAC1* mRNA.
(TIF)

Figure S3 Wild-type and *SCS3 YFT2* gene-deletion strains exhibit comparable lipid droplet phenotypes under various stress conditions. A. The indicated strains were grown in inositol-free media supplemented with 10 µM or 100 µM inositol (top and bottom panels, respectively). Early log phase and saturated overnight cultures were analyzed for lipid droplets by direct staining of live cells with BODIPY 493/503 as described in Materials and Methods. B. Strains were grown in oleate-containing YPO media and stained for lipid droplets as in panel A. C. Effect of the *sec13-1* mutation on lipid droplet production in wild-type and *SCS3 YFT2* gene-deletion strains. SGA methodology was used to introduce the conditionally-viable *sec13-1* mutation as in Figure 9a. Haploid progeny obtained at the permissive temperature of 22°C were grown to early log phase in haploid selection media, fixed, stained and imaged for lipid droplets as described in Figure 2b.
(TIF)

Figure S4 Comparisons of genetic interaction scores between genes identified in two or more screens. Epsilon scores are compared for 167 (A) and 72 (B) strains that yielded genetic interactions with the *SCS3 YFT2* double gene-deletion strain and the *SCS3* or *YFT2* single gene-deletion strains, respectively. The data are represented in Figure 3b and resulted from interactions that, in all cases, showed size differences of >40 pixels between query and control screens and a *p* value<0.01.
(TIF)

Figure S5 Correlation of genetic interaction scores between dubious ORFs and their genomic neighbors. Deletion strains corresponding to dubious ORFs in SGD that were contained in the set of 636 *SCS3* and/or *YFT2* genetic interactions (i.e. satisfied our stringent criteria for pixel size, p value and ε score) were manually examined in GBrowse for overlap with the coding and likely promoter regions of verified genes. From the resulting 40 dubious ORFs and their neighboring/overlapping verified gene-deletions we performed pairwise comparisons of the ε values obtained in *scs3*Δ, *yft*2Δ and *scs3*Δ *yft*2Δ screens (i.e. 120 ε values) and determined a Pearson correlation coefficient of 0.75.
(TIF)

Figure S6 Lipid profiling of wild-type and *SCS3/YFT2* gene-deletion strains. A. Neutral lipid content is comparable between a wild-type strain and strains deleted for *SCS3* and/or *YFT2*. Log phase cells were metabolically labeled for 2 hours with ^14^C-acetate and lipids were extracted by the two-step 4°C method [51]. Neutral lipids were separated by one-dimensional TLC in hexanes∶ethyl ether∶acetic acid (80∶20∶1), detected by phosphorimage and analysed with ImageQuant software. The TLC is representative of three independent labeling experiments. B. Deletion of *SCS3* and *YFT2* does not affect total cellular lipid profiles. Lipids from log phase wild-type and *scs3*Δ *yft2*Δ cells were prepared from unlabeled log phase cells as described above and analyzed by quantitative mass spectrometry at the Kansas Lipidomics Research Center Analytical Laboratory. The abundance of lipid species is graphed as nmol/10^8^ cells. Left panel: total polar lipids, right panel: total neutral lipid species. Error bars indicate the standard deviation from three biological replicate analyses. Lysophosphatidic acid, LPA; lysophosphatidylethanolamine, LPE; phosphatidic acid, PA; phosphatidylcholine, PC; phosphatidylethanolamine, PE; phosphatidylglycerol, PG; phosphatidylinositol, PI; phosphatidylserine, PS; diacylglycerol, DAG; triaclyglycerol, TG.
(TIF)

Figure S7 CPY processing and tunicamycin sensitivity of *SCS3/YFT2* gene deletion strains. A CPY processing in wild-type *and scs3*Δ *yft*2Δ strains. Strains were transformed with a *MET15* gene-containing plasmid prior to growth in methionine-minus SC media. Cells were pulse-labeled with Tran ^35^S-labeling reagent for 10 mins and chased with excess cold methionine and cysteine as depicted. Extract preparation and immuoprecipitation were as described [104]. CPY immunoprecipitates were separated on 8% SDS-polyacrylamide gels and visualized by autoradiography. Migration of ER-glycosylated (*p1*) and Golgi-modified (*p2*) precursors and vacuolar mature form (*mCPY*) are indicated. B Tunicamycin hypersensitivity of *SCS3* and *YFT2* deletion strains. Two-fold serial dilutions of the indicated strains were spotted onto SC media containing 100 µM inositol with or without 1.0 µg/ml tunicamycin. Plates were photographed at 2 and 3 days respectively.
(TIF)

Figure S8 Attenuation of the UPR in *SCS3 YFT2* gene-deletion strains. Northern analysis of UPR induction after DTT treatment. Cells were grown to early log phase at 30°C in synthetic complete media, treated with 6 mM DTT for 1 hour and either left in DTT-containing media (panel A) or washed into fresh 30°C media lacking DTT (panel B). Cells were harvested over the time courses indicated. *HAC1* mRNA (reporting both unspliced Hac1^u^ and spliced Hac1^i^ forms) was detected as described in Figure 7c. The extent of Hac1 splicing is expressed as % of total Hac1 mRNA and is indicated under each lane.
(TIF)

Table S1 Stringent Set of *SCS3* and *YFT2* Genetic Interactions.
(XLSX)

Table S2 GOSlim Component Analysis of *SCS3* and *YFT2* Gene Interactions.
(XLS)
