# Supplementary figures and images for: SCS3 and YFT2 Link Transcription of Phospholipid Biosynthetic Genes to ER Stress and the UPR
Source: PLoS Genet. 2012 Aug 23;8(8):e1002890. doi: 10.1371/journal.pgen.1002890 (PMC3426550; doi:10.1371/journal.pgen.1002890)

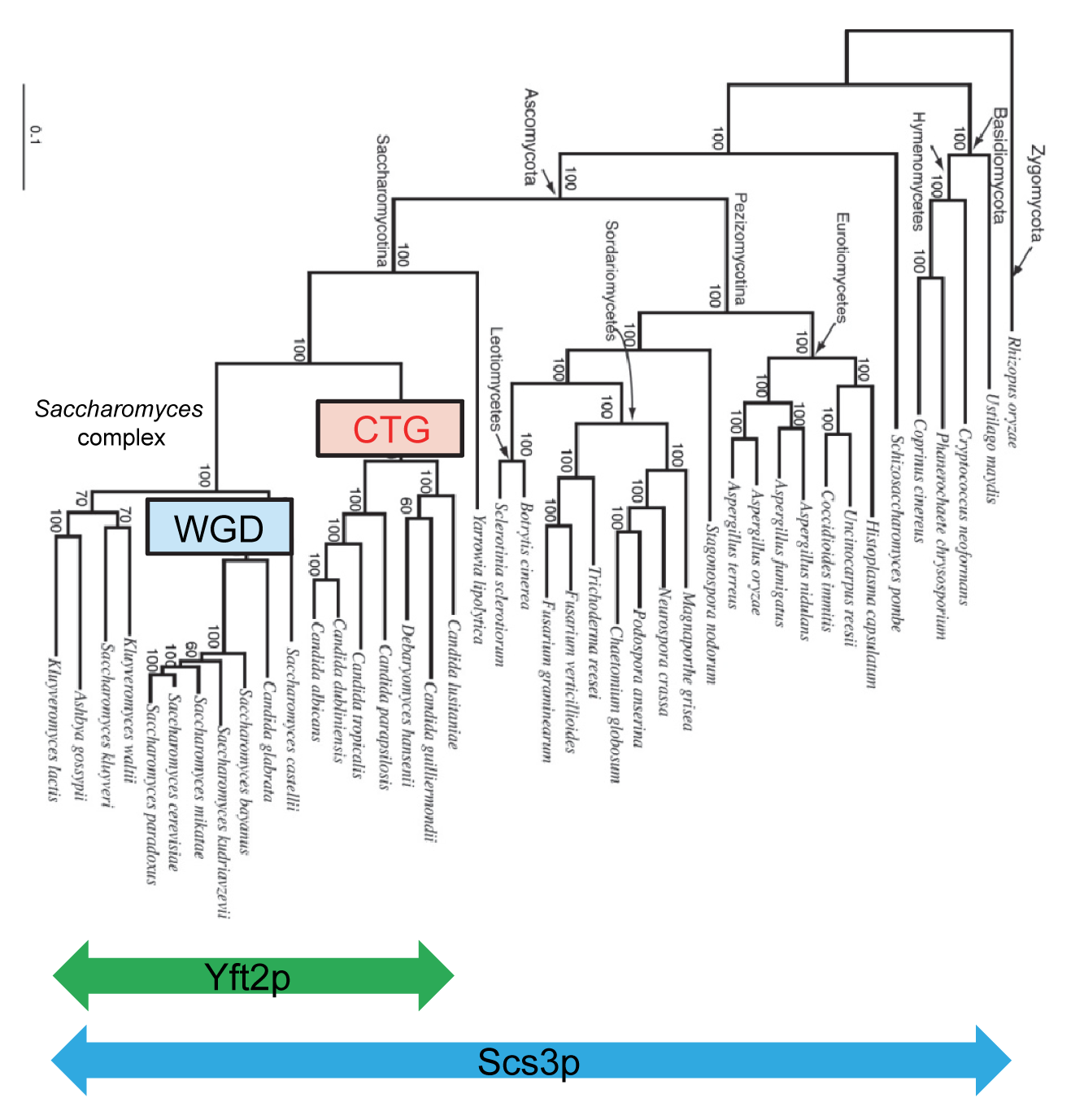

Supplement: Figure S1 — Phylogenetic distribution of SCS3 and YFT2 in the genomes of sequenced fungi. Evolutionary relationships were constructed using the sequences of 153 genes that are universally present in the 42 genomes shown [26]. Major clades are indicated, including the Saccharomyces complex, the group of species that share the whole-genome duplication (WGD) and those with the variant genetic code (CTG). YFT2 and SCS3 orthologs were identified by Blast searches of yeast genomes at NCBI or the Broad Institute databases using the complete amino acid sequences for SCS3 or YFT2 or the corresponding signature sequences from the fourth transmembrane domain of each protein. FIT gene orthologs were identified in all organisms except P. chrysosporium, A. terreus and C. globosum. Figure was adapted from ref. [26]. (TIF) [file pgen.1002890.s001.tif]

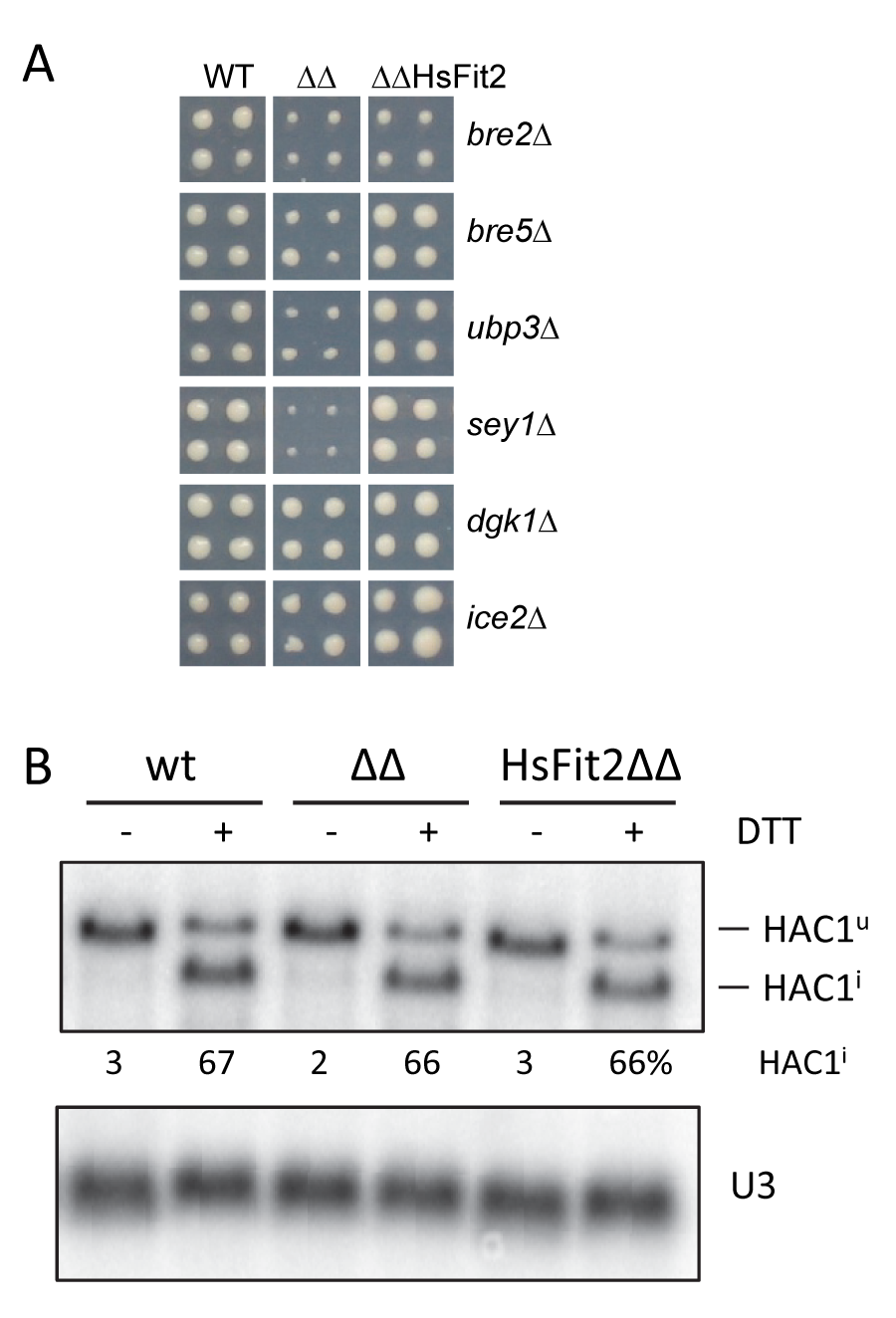

Supplement: Figure S2 — Expression of HsFit2 in SCS3 YFT2 gene-deletion strains. A HsFit expression complements synthetic growth defects in scs3Δ yft2Δ xxxΔ strains. The HsFit2 gene, controlled by a TDH3 promoter and a CYC1 terminator, was substituted for YFT2 by one-step gene replacement in the Y7092 scs3Δ::URA3 query strain. SGA techniques were then used to recover wild-type, scs3Δ yft2Δ and HsFit2-expressing scs3Δ yft2Δ strains containing the indicated deletions from the viable gene-deletion array. Final haploid colonies generated in quadruplicate were printed onto SGA selection media and photographed at 48 hours. Each row of panels is deleted for a different array gene (indicated on the right). Wild-type (WT; SCS3 YFT2), scs3Δ yft2Δ (ΔΔ) and HsFit2-expressing scs3Δ yft2Δ (ΔΔ HsFit2) strains are annotated across the top. Note that only the ice2Δ strain is an inositol auxotroph and the growth phenotypes were assayed on SGA medium which contains excess inositol. B HsFit expression in the scs3Δ yft2Δ strain does not induce the UPR. Northern analysis of the distribution of HAC1 mRNA into unspliced Hac1u and spliced Hac1i forms and stable U3 snRNA was performed on RNA from early log phase cultures of wild-type, scs3Δ yft2Δ (ΔΔ) and HsFit2-expressing scs3Δ yft2Δ (ΔΔ HsFit2) strains grown in the presence of excess inositol. The extent of Hac1 splicing is expressed as % of total Hac1 mRNA and is indicated under each lane. ER stress in untreated and DTT-treated cells is reported by the accumulation of spliced HAC1 mRNA. (TIF) [file pgen.1002890.s002.tif]

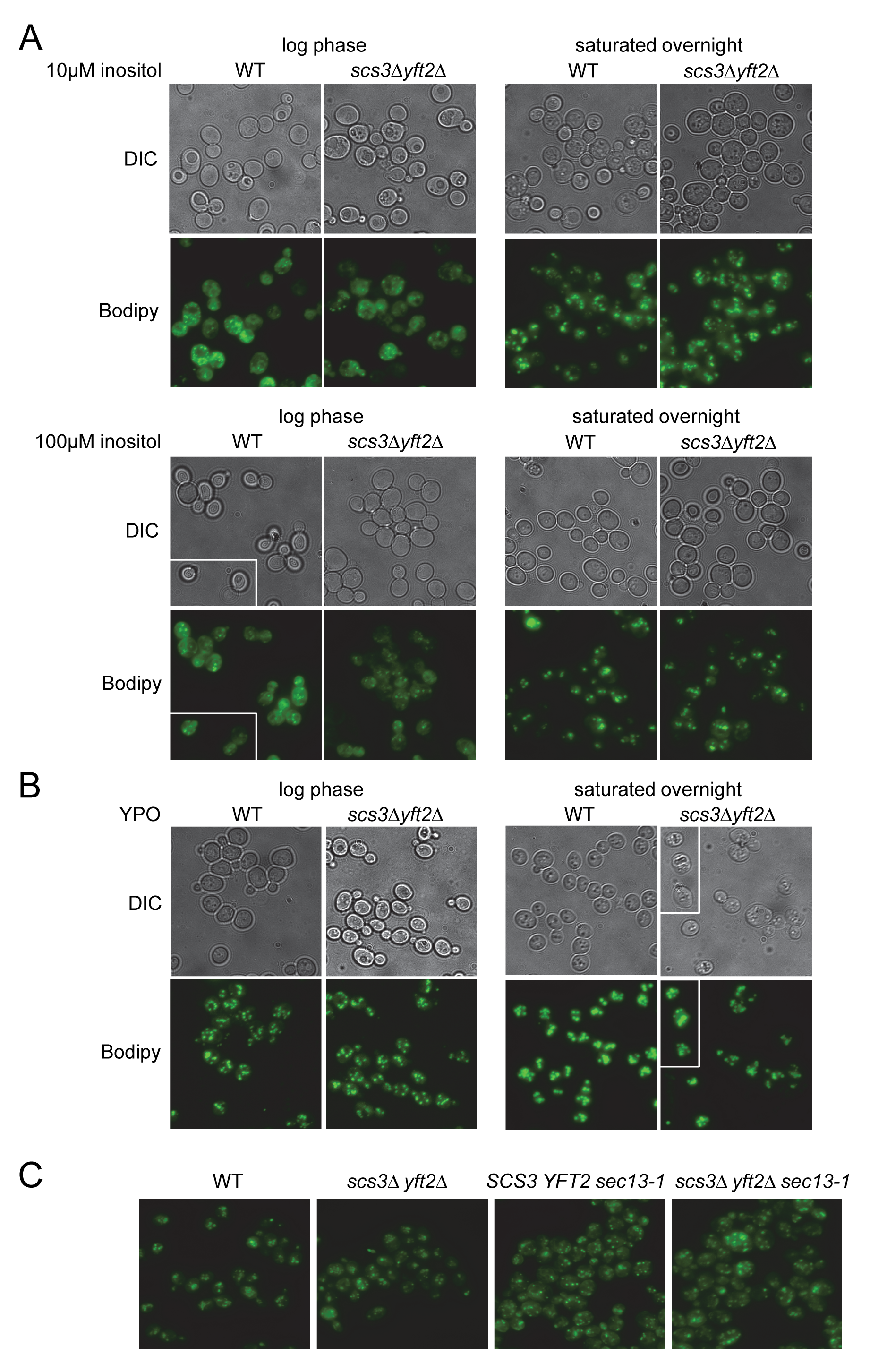

Supplement: Figure S3 — Wild-type and SCS3 YFT2 gene-deletion strains exhibit comparable lipid droplet phenotypes under various stress conditions. A. The indicated strains were grown in inositol-free media supplemented with 10 µM or 100 µM inositol (top and bottom panels, respectively). Early log phase and saturated overnight cultures were analyzed for lipid droplets by direct staining of live cells with BODIPY 493/503 as described in Materials and Methods. B. Strains were grown in oleate-containing YPO media and stained for lipid droplets as in panel A. C. Effect of the sec13-1 mutation on lipid droplet production in wild-type and SCS3 YFT2 gene-deletion strains. SGA methodology was used to introduce the conditionally-viable sec13-1 mutation as in Figure 9a. Haploid progeny obtained at the permissive temperature of 22°C were grown to early log phase in haploid selection media, fixed, stained and imaged for lipid droplets as described in Figure 2b. (TIF) [file pgen.1002890.s003.tif]

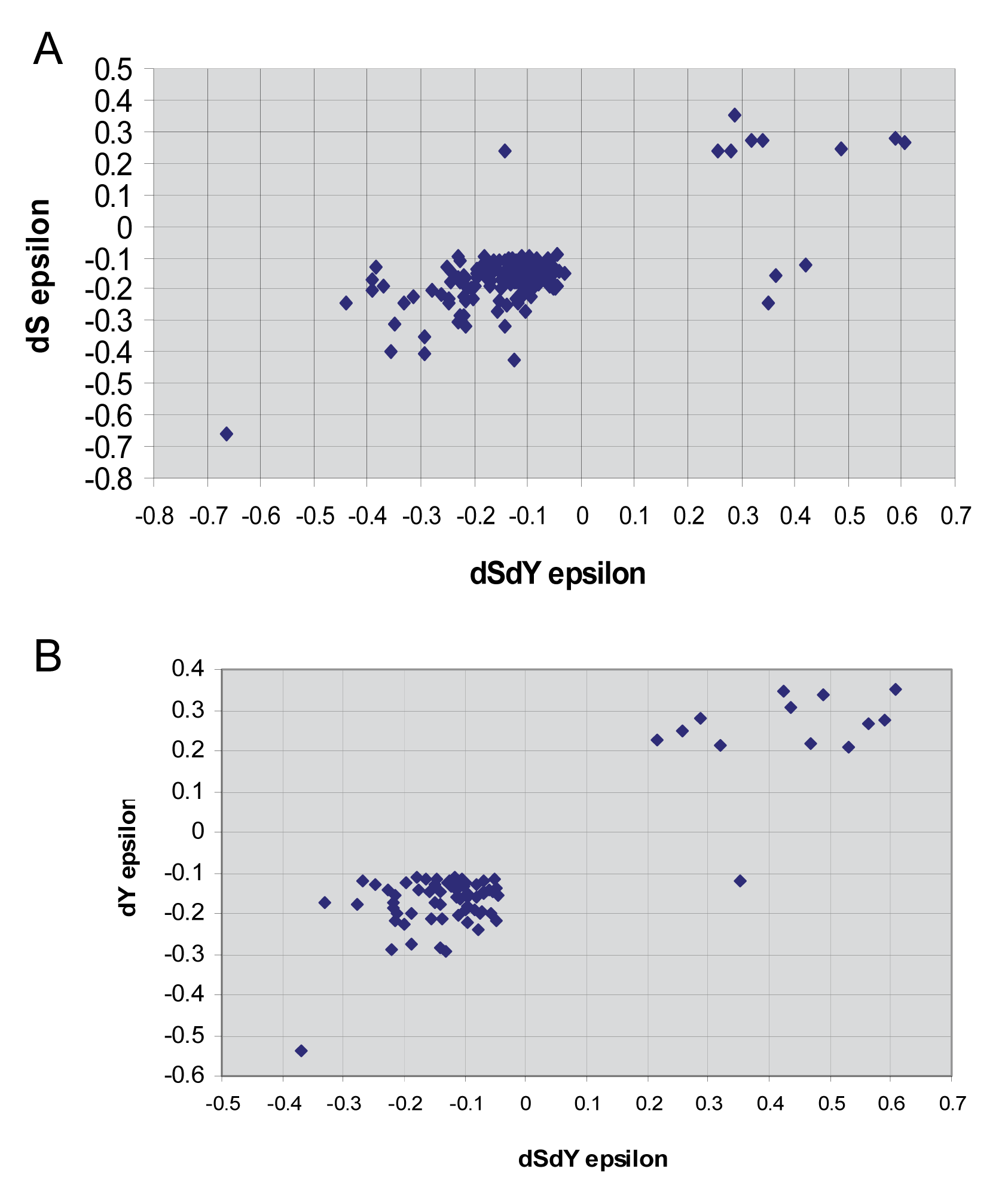

Supplement: Figure S4 — Comparisons of genetic interaction scores between genes identified in two or more screens. Epsilon scores are compared for 167 (A) and 72 (B) strains that yielded genetic interactions with the SCS3 YFT2 double gene-deletion strain and the SCS3 or YFT2 single gene-deletion strains, respectively. The data are represented in Figure 3b and resulted from interactions that, in all cases, showed size differences of >40 pixels between query and control screens and a p value<0.01. (TIF) [file pgen.1002890.s004.tif]

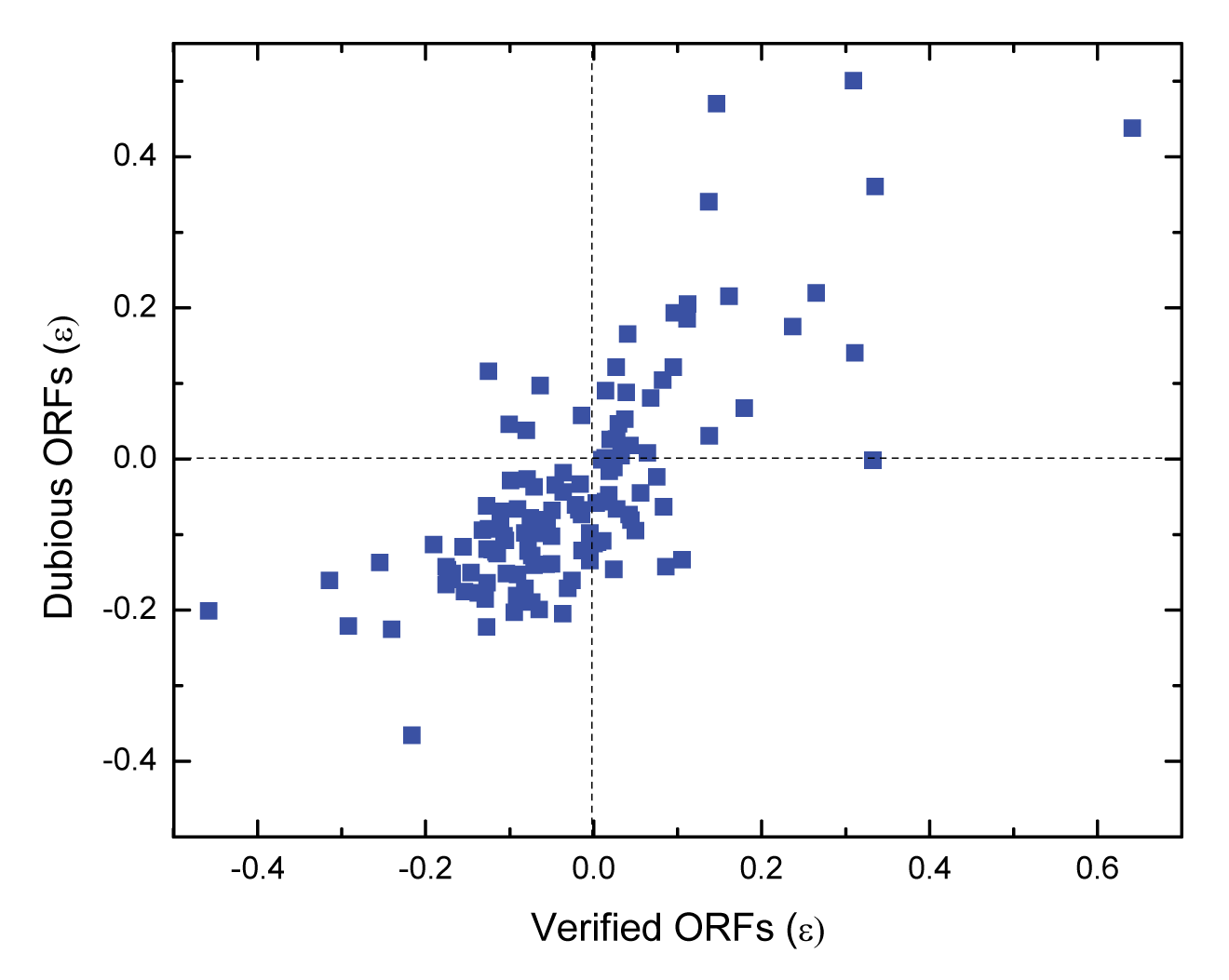

Supplement: Figure S5 — Correlation of genetic interaction scores between dubious ORFs and their genomic neighbors. Deletion strains corresponding to dubious ORFs in SGD that were contained in the set of 636 SCS3 and/or YFT2 genetic interactions (i.e. satisfied our stringent criteria for pixel size, p value and ε score) were manually examined in GBrowse for overlap with the coding and likely promoter regions of verified genes. From the resulting 40 dubious ORFs and their neighboring/overlapping verified gene-deletions we performed pairwise comparisons of the ε values obtained in scs3Δ, yft2Δ and scs3Δ yft2Δ screens (i.e. 120 ε values) and determined a Pearson correlation coefficient of 0.75. (TIF) [file pgen.1002890.s005.tif]

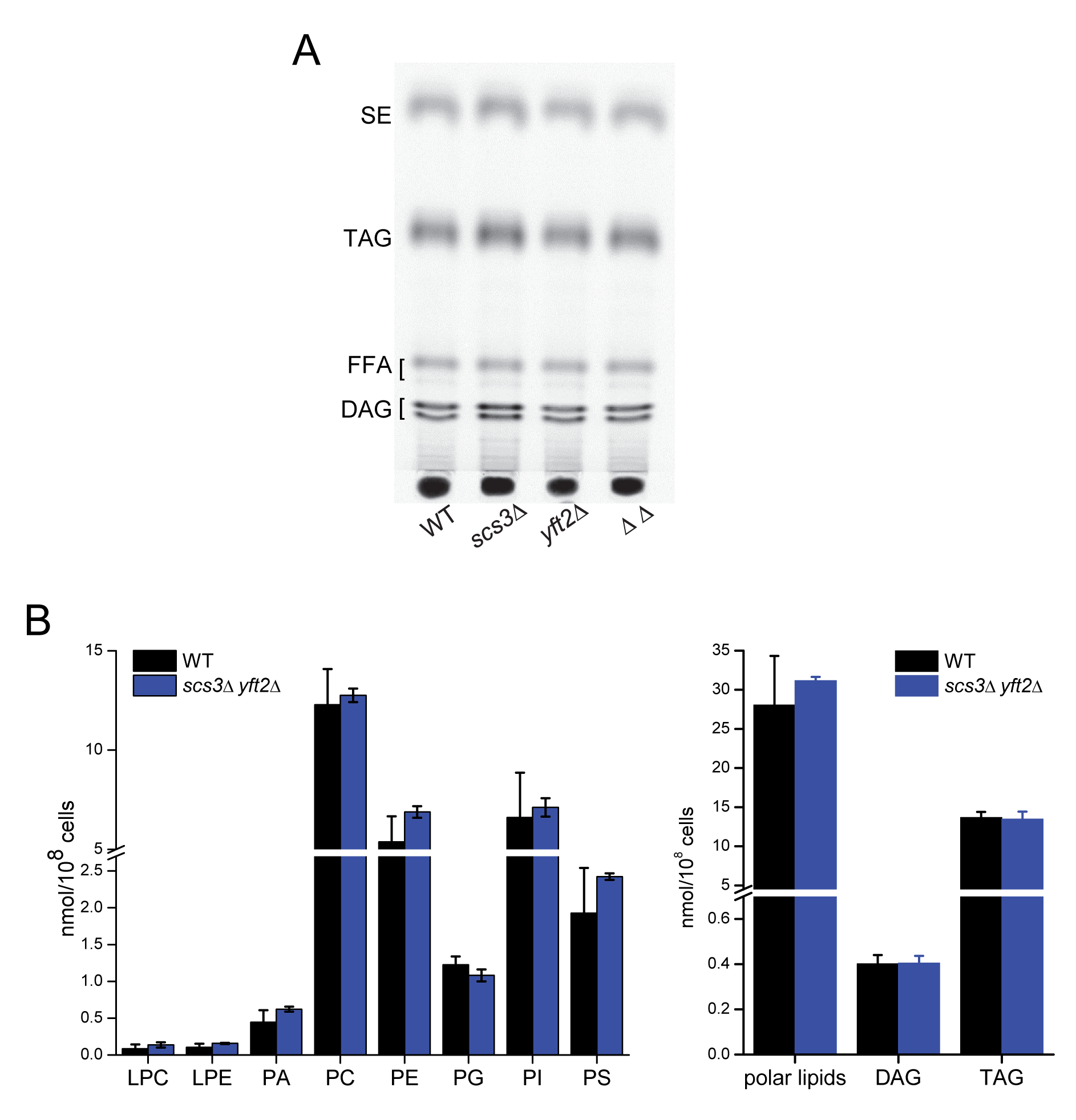

Supplement: Figure S6 — Lipid profiling of wild-type and SCS3/YFT2 gene-deletion strains. A. Neutral lipid content is comparable between a wild-type strain and strains deleted for SCS3 and/or YFT2. Log phase cells were metabolically labeled for 2 hours with 14C-acetate and lipids were extracted by the two-step 4°C method [51]. Neutral lipids were separated by one-dimensional TLC in hexanes∶ethyl ether∶acetic acid (80∶20∶1), detected by phosphorimage and analysed with ImageQuant software. The TLC is representative of three independent labeling experiments. B. Deletion of SCS3 and YFT2 does not affect total cellular lipid profiles. Lipids from log phase wild-type and scs3Δ yft2Δ cells were prepared from unlabeled log phase cells as described above and analyzed by quantitative mass spectrometry at the Kansas Lipidomics Research Center Analytical Laboratory. The abundance of lipid species is graphed as nmol/108 cells. Left panel: total polar lipids, right panel: total neutral lipid species. Error bars indicate the standard deviation from three biological replicate analyses. Lysophosphatidic acid, LPA; lysophosphatidylethanolamine, LPE; phosphatidic acid, PA; phosphatidylcholine, PC; phosphatidylethanolamine, PE; phosphatidylglycerol, PG; phosphatidylinositol, PI; phosphatidylserine, PS; diacylglycerol, DAG; triaclyglycerol, TG. (TIF) [file pgen.1002890.s006.tif]

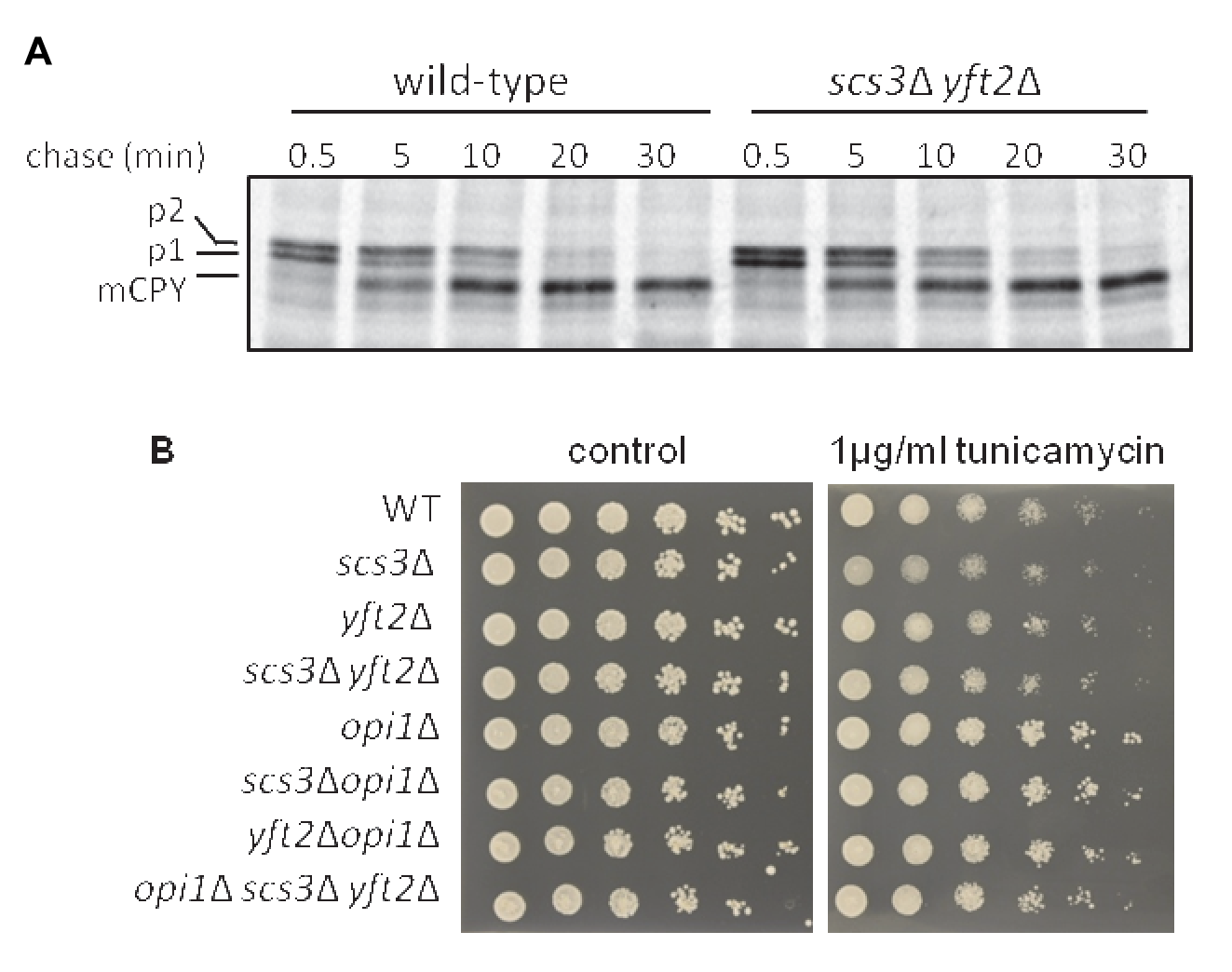

Supplement: Figure S7 — CPY processing and tunicamycin sensitivity of SCS3/YFT2 gene deletion strains. A CPY processing in wild-type and scs3Δ yft2Δ strains. Strains were transformed with a MET15 gene-containing plasmid prior to growth in methionine-minus SC media. Cells were pulse-labeled with Tran 35S-labeling reagent for 10 mins and chased with excess cold methionine and cysteine as depicted. Extract preparation and immuoprecipitation were as described [104]. CPY immunoprecipitates were separated on 8% SDS-polyacrylamide gels and visualized by autoradiography. Migration of ER-glycosylated (p1) and Golgi-modified (p2) precursors and vacuolar mature form (mCPY) are indicated. B Tunicamycin hypersensitivity of SCS3 and YFT2 deletion strains. Two-fold serial dilutions of the indicated strains were spotted onto SC media containing 100 µM inositol with or without 1.0 µg/ml tunicamycin. Plates were photographed at 2 and 3 days respectively. (TIF) [file pgen.1002890.s007.tif]

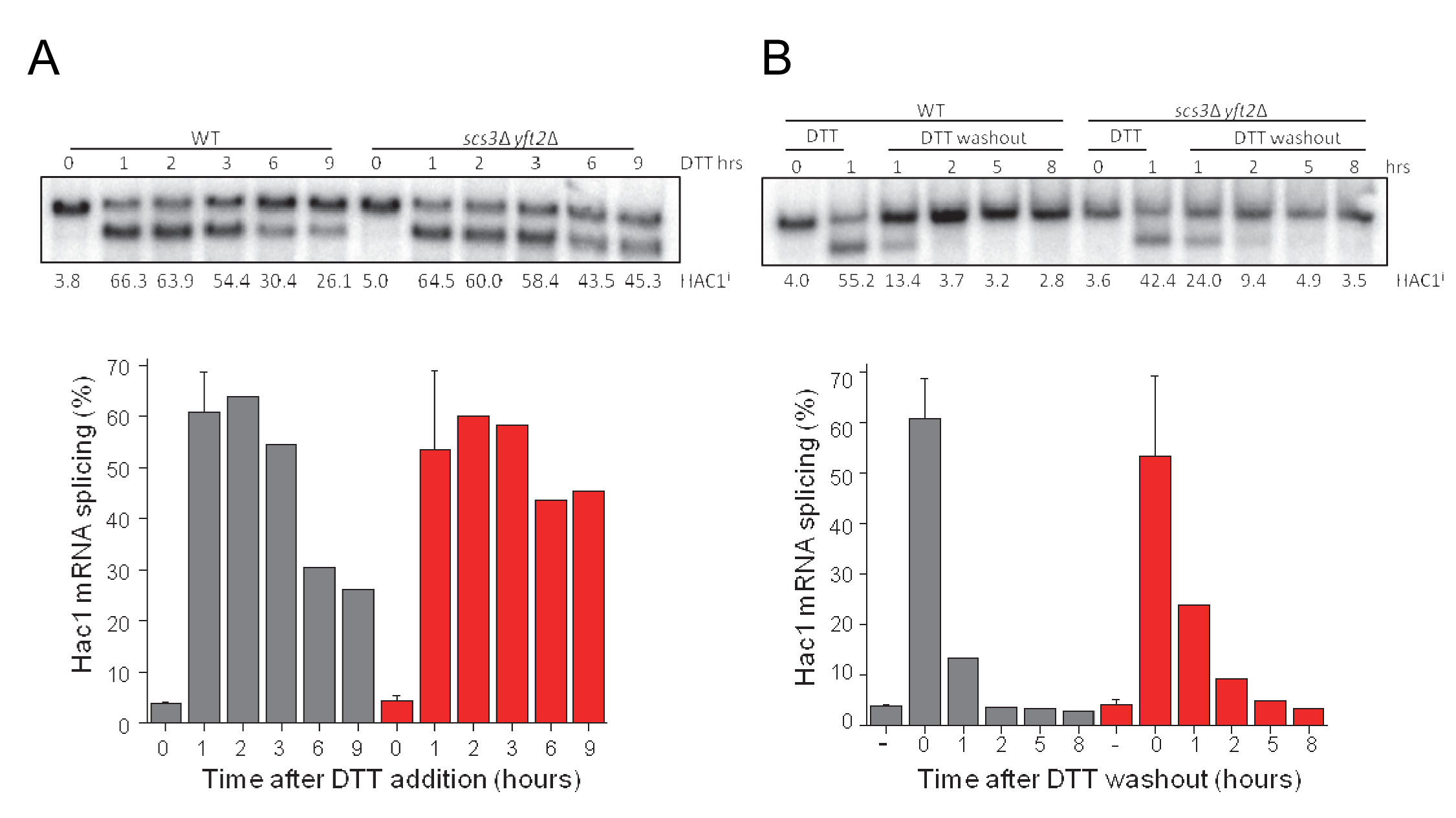

Supplement: Figure S8 — Attenuation of the UPR in SCS3 YFT2 gene-deletion strains. Northern analysis of UPR induction after DTT treatment. Cells were grown to early log phase at 30°C in synthetic complete media, treated with 6 mM DTT for 1 hour and either left in DTT-containing media (panel A) or washed into fresh 30°C media lacking DTT (panel B). Cells were harvested over the time courses indicated. HAC1 mRNA (reporting both unspliced Hac1u and spliced Hac1i forms) was detected as described in Figure 7c. The extent of Hac1 splicing is expressed as % of total Hac1 mRNA and is indicated under each lane. (TIF) [file pgen.1002890.s008.tif]
